# Molecular characterization of *Streptococcus suis* isolates recovered from diseased pigs in Europe

**DOI:** 10.1186/s13567-024-01366-y

**Published:** 2024-09-27

**Authors:** Kevin Li, Sonia Lacouture, Eric Lewandowski, Eric Thibault, Hubert Gantelet, Marcelo Gottschalk, Nahuel Fittipaldi

**Affiliations:** 1 Groupe de recherche sur les maladies infectieuses en production animale, and Centre de recherche en infectiologie porcine et avicole, Faculté de médecine vétérinaire, Université de Montréal, St-Hyacinthe, QC Canada; 2 Ceva Biovac, Beaucouzé, France

**Keywords:** Swine infectious diseases, *Streptococcus suis*, genomic epidemiology, antimicrobial resistance, virulence-associated genes, Europe

## Abstract

*Streptococcus suis* is a major swine pathogen and zoonotic agent, causing important economic losses to the porcine industry. Here, we used genomics approaches to characterize 251 *S. suis* isolates recovered from diseased pigs across Belgium, France, Germany, Hungary, the Netherlands, Spain, and the United Kingdom. We identified 13 serotypes, being serotypes 9 and 2 the most prevalent, and 34 sequence types (STs), including 16 novel STs, although ST16 and ST1 dominated the strain population. Phylogenetic analysis revealed complex genetic relationships, notable geographic clustering, and potential differential capacity for capsular switching among serotype 9 isolates. We found antimicrobial resistance (AMR) genes in 85.3% of the isolates, with high frequencies of genes conferring resistance to tetracyclines and macrolides. Specifically, 49.4% of the isolates harbored the *tetO* gene, and 64.9% possessed the *ermB* gene. Additionally, we observed a diverse array of virulence-associated genes (VAGs), including the classical VAGs *mrp*, *epf*, and *sly*, with variable presence across different genotypes. The high genetic diversity among European *S. suis* isolates highlights the importance of targeted antimicrobial use and flexible vaccine strategies. Rapid strain characterization is crucial for optimizing swine health management, enabling tailored interventions like the development of autovaccines to mitigate *S. suis* infections.

**Supplementary Information:**

The online version contains supplementary material available at 10.1186/s13567-024-01366-y.

## Introduction

*Streptococcus suis* is a swine pathogen responsible for important economic losses to the porcine industry [1]. The organism can asymptomatically colonize the upper respiratory tract of swine and can also cause several major diseases in pigs, including sepsis, meningitis, arthritis, endocarditis, and sudden death [1–3]. *S. suis* is also an emerging zoonotic agent, capable of transmission to individuals who come into close contact with infected pigs, handle pork or pork by-products, or, particularly in East and Southeast Asia, consume dishes containing raw pork or pork blood [4–8].

Serotyping continues to be the main *S. suis* typing scheme. Based on an antigenic reaction directed against the capsular polysaccharide (CPS), 35 different *S. suis* serotypes were initially recognized, although 6 of them were later ascribed to other bacterial species [9–11]. Thus, the organism currently comprises 29 serotypes. However, this number does not account for several novel capsular types that have recently been recognized using molecular methods [12–17]. In addition to serotyping, other typing schemes such as multilocus sequence typing (MLST) are used to characterize *S. suis* isolates [18], and have been instrumental in discovering a relatively high genetic diversity of the species, with more than 2800 described sequence types (STs) [19, 20].

Although the prevalence of *S. suis* serotypes and genotypes varies among different geographical locations and time periods, strains of ST1 serotype 2 and ST16 serotype 9 are frequently recovered from diseased pigs in Europe [11]. Additionally, strains of other serotypes and genetic lineages, such as ST1552 serotype 1 and ST29 serotype 7, have also been associated with swine diseases in Europe [21–25]. *S. suis* has been proposed to act as an important reservoir for antimicrobial resistance (AMR) genes, with implications for both swine and public health [26]. Several reports from Europe that characterized *S. suis* AMR have noted a high prevalence of resistance to tetracyclines, macrolides, and lincosamides, as well as to other antimicrobials, raising concerns about the role of *S. suis* in AMR dissemination [21, 27–33]. However, important knowledge gaps remain, highlighting the need for further characterization of a broader range of isolates from Europe that better represents the extent of *S. suis* genetic diversity.

The virulence of *S. suis* is multifactorial, and is influenced by a combination of both host and pathogen traits [1, 34]. The CPS, which shields the bacterium from immune recognition and clearance, is considered the primary virulence factor [34, 35]. However, a wide array of additional factors has been linked to *S. suis* virulence, including many factors whose role(s) in the pathogenesis of infection remain debatable [34, 35]. Indeed, more than 80 virulence-associated genes (VAGs) have been described as important for the virulence of the organism. These include traditional VAGs such as *mrp* (encoding a muramidase-released protein), *epf* (an extracellular factor), and *sly* (the hemolysin known as suilysin), as well as several VAGs whose roles and prevalence across different *S. suis* genotypes are not yet fully understood [34–41].

In response to increasing concerns about AMR and to address gaps in knowledge regarding the prevalence of virulence-associated genes (VAGs) among strains circulating in Europe, here we characterized using genomics 251 *S. suis* isolates recovered from diseased pigs across seven European countries. Our findings reveal substantial genetic diversity among these isolates, which displayed a broad spectrum of AMR genes and VAGs.

## Materials and methods

### Isolate collection, DNA extraction, and serotyping

We used a convenience sample comprising 251 *S. suis* isolates recovered between years 2012 to 2020 from diseased pigs in France (*N* = 138), the Netherlands (*N* = 62), Germany (*N* = 27), and the United Kingdom (*N* = 15); a few isolates from Belgium (*N* = 5), Hungary (*N* = 2) and Spain (*N* = 2) were also included (Additional file 1). The majority of these isolates had been used or considered for use in autovaccine formulations, providing a representative snapshot of the prevalent field strains responsible for infections across these regions. For molecular testing and genome sequencing, strains were grown overnight in Todd-Hewitt broth (BD Bioscience, San Jose, CA, USA) supplemented with 0.2% yeast extract, at 37 °C under 5% CO_2_. DNA was prepared from 5 mL of these cultures using the QIAamp DNA minikit (Qiagen, Toronto, Canada) according to the manufacturer’s protocol for Gram-positive bacteria. The serotype of the isolates was determined using a multiplex PCR assay detecting CPS biosynthesis genes, as previously described [42]. Briefly, each PCR consisted of template DNA, a particular primer mix, and PCR master mix (Multiplex PCR Mastermic, Qiagen). PCR products were resolved on 2% agarose gel stained with SYBR safe (ThermoFisher) and visualized under UV light. Serotypes were determined accordingly to the DNA band position, as described [42]. Differentiation of serotype pairs 1 and 14, as well as 2 and 1/2, cannot be achieved using this PCR assay. To resolve these pairs, we used genome-based in silico PCR, which is further described below.

### Whole-genome sequencing and bioinformatics analysis

Sequencing libraries were prepared using Nextera XT kits (Illumina, San Diego, CA, USA) following the manufacturer’s instructions. Libraries were sequenced on an iSeq 100 instrument (Illumina). For approximately half of the isolates, libraries were sequenced as paired-end reads of 150 + 150 bp, while the other half were sequenced as paired-end reads of 300 + 300 bp. Both datasets were compatible for all downstream analyses, and the variability in read lengths was solely due to the availability of reagents in our laboratories.

Serotypes and MLST profiles were determined directly from the short-read genome data using a previously described in silico *S. suis* serotyping pipeline that uses read alignment to a custom database of serotype-specific *cps* locus sequences, and which can discriminate between serotype pairs 1 and 14, as well as 2 and 1/2, based on a single nucleotide polymorphism (SNP) in the gene *cpsK* [19]. In cases where low coverage prevented the automated determination of a serotype by the pipeline, we manually inspected intermediate files in the pipeline output, namely the “score” file generated by the pipeline was manually reviewed. A serotype was then assigned if: 1) the “score” file reported a low depth of coverage serotype, and it matched the PCR results or 2) the “score” file identified a serotype in a PCR “untypable” result. When manual inspection revealed a blank score file, the isolate was deemed to be untypable. For MLST determinations, we used the PubMLST *S. suis* MLST database [20], downloaded on June 10^th^, 2024. Using standard nucleotide BLAST [43], we also interrogated the genomes for the presence of the 3 classical VAGs, namely genes *mrp*, *epf*, and *sly*, as well as of 84 additional putative virulence factors [34, 36]. We also examined the presence of 26 putative zoonotic virulence factors (PZVFs), nine of which were included among the abovementioned set of 84 VAGs [40]. To that end, we first performed de novo genome assemblies using the A5-MiSeq pipeline [44] and annotated the resulting contigs with Prokka [45]. To investigate AMR gene content, we used the read mapping-based tool SRST2 [46] and the Comprehensive Antibiotic Resistance Database (CARD) v3.0.8 [47]. Visualizations were generated using the base R software version 4.2.2 [48] and edited in Adobe Illustrator.

### Phylogenetic analysis

Core genome-based phylogenetic analysis was performed as follows: using the Snippy algorithm [49], we first identified SNPs among the 251 *S. suis* isolates in our collection relative to the genome sequences of the ST1 serotype 2 reference strain P1/7 (GenBank accession number NC_012925.1). For confirmation of phylogenetic findings, a separate analysis was performed using the ST16 serotype 9 strain GD-0088 (GenBank accession number NZ_LR738723.1) as reference. Identified SNPs were next reduced to the core SNPs set using the Snippy-core function [49]. Maximum likelihood phylogenetic trees were then constructed with FastTree 2.1.10 [50] with 1000 bootstrap replications, and visualized and annotated using R [48], and the R library ggtree [51].

## Results

### High serotype diversity with dominance of serotypes 9 and 2 among European *S. suis* isolates

**Figure 1:**
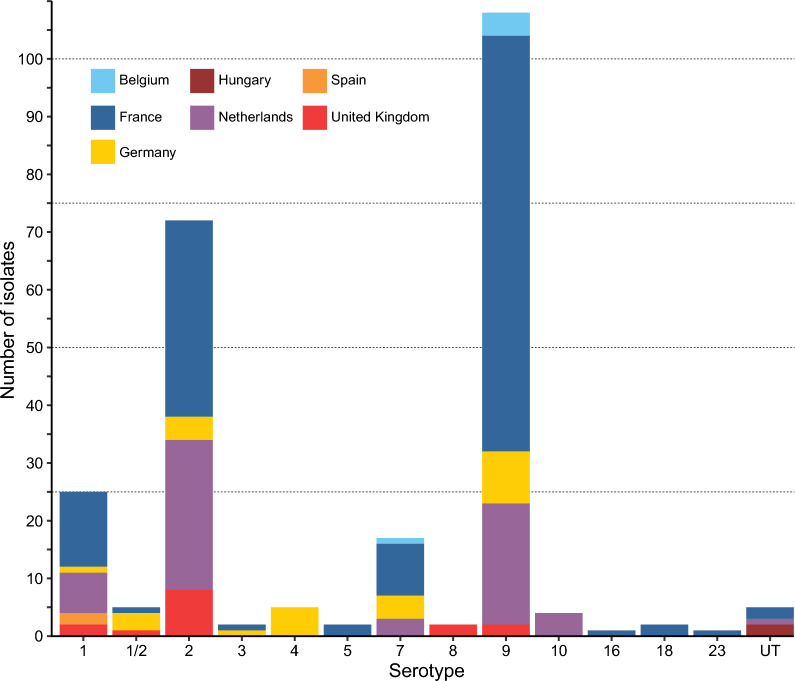
**Serotype distribution of *S. suis* isolates across seven European countries.** Serotypes 2, 9, 1 and 7 dominated the population and there were important differences in distribution patterns across the different countries. Each country is represented by a distinct color, as indicated by the color-coded key on the chart.

To identify the serotype of the *S. suis* isolates, we used both multiplex PCR and genomic based in silico approaches. We identified 13 serotypes among the 251 isolates. Consistent with previous reports from Europe [24], most isolates were of serotype 9 (*N* = 108/251, 43.0%), followed by those of serotype 2 (*N* = 72/251, 28.7%). The remaining isolates were distributed among serotypes 1 (*N* = 25) and 7 (*N* = 17), 4 and 1/2 (*N* = 5 each), 10 (*N* = 4), 5 (*N* = 2), 3 (*N* = 2), 18 (*N* = 2), 8 (*N* = 2), 16 (*N* = 1), and 23 (*N* = 1) (Figure 1). Five isolates were untypable. Considering isolates of serotype 2 and 1/2 together (as these serotypes cannot be resolved by the multiplex PCR), there was a 100% agreement between results from the multiplex PCR and genomics-based in silico serotyping, although this required manual verification of in silico results for 35 isolates with low read depth of coverage (see Materials and methods).

### Geographic variation in serotype distribution of *S. suis* isolates across Europe

Serotype 9 predominated among isolates recovered in France (Figure 1), accounting for 52.2% of the isolates from that country (72 out of 138), followed by the Netherlands with 33.9% (21 out of 62), Germany with 33.3% (9 out of 27), and the United Kingdom with 13.3% (2 out of 15). In comparison, serotype 2 isolates were recovered from four countries. The United Kingdom had the highest proportion of serotype 2 strains at 53.3% (8 out of 15), followed by the Netherlands at 43.5% (27 out of 62), France at 24.6% (34 out of 138), and Germany at 14.8% (4 out of 27).

Isolates of serotype 1 were mostly from France and the Netherlands, although they were also found in the United Kingdom, Spain, and Germany. Most serotype 7 isolates originated from France, Germany, and the Netherlands, with one isolate from Belgium. Germany and France had isolates of serotype 3 and 1/2, with the latter also found in the United Kingdom. Isolates of serotypes 4, 8, and 10 were exclusively found in Germany, the United Kingdom, and the Netherlands, respectively, while France was the only country with isolates of serotypes 5, 16, 18, and 23.

It should be noted that Belgium, Spain, and Hungary had limited representation in our collection. In Belgium, out of five isolates, four were of serotype 9 and one of serotype 7; the two Spanish isolates were of serotype 1; and the two isolates from Hungary were untypable. Additionally, three other untypable isolates were identified: two from France and one from the Netherlands. Inspection of the de novo genome assemblies of these isolates revealed that the Hungarian isolates have lost several *cps* genes. However, remnant *cps* sequences appeared to indicate they originally possessed a *cps2* locus (Additional file 2A). Similarly, manual inspection of the *cps* loci of the three other untypable isolates revealed the absence of several key *cps* genes (Additional files 2B-D).

### MLST identifies dominant and several novel genotypes among European *S. suis* isolates

We next acquired MLST profiles for the *S. suis* isolates directly from the short-read genome data and identified 34 different STs, including 28 isolates (11.2%) with 16 novel unique allele combinations of the 7 housekeeping genes used in the *S. suis* MLST scheme (Additional file 3). The allelic profiles for these novel STs (ST2753, ST2757, ST2760, ST2767-2769, ST2771-2775, ST2790, ST2791, ST2793, ST2796, and ST2798) have been deposited in the *S. suis* PubMLST database [20]. Most serotype 2 and serotype 9 isolates belonged to ST1 (*N* = 49) and ST16 (*N* = 75), respectively. Serotype 2 isolates were also classified into 7 additional STs, including STs clonally related to ST1 such as ST2 (*N* = 5), ST2753 (*N* = 3) and ST2768 (*N* = 2), as well as genetically distant ST28 (*N* = 11) and ST20 (*N* = 2). We observed higher genetic diversity among the 108 serotype 9 isolates, with 11 STs other than ST16 represented. These included ST16 derivatives such as ST1520 (*N* = 4), along with 3 novel ST16 derivatives: ST2760 (*N* = 2), ST2772 (*N* = 2), ST2773 (*N* = 1). Genetically unrelated serotype 9 genotypes included ST1508 (*N* = 6), ST147 (*N* = 3), and ST819 (*N* = 2), which are clonally related to ST14 (not detected in our study). Another genetically distant serotype 9 genotype, ST1521 (*N* = 7), was also identified. The remaining 6 serotype 9 isolates belonged to 3 novel STs ST2757 (*N* = 4), ST2767 (*N* = 1), and ST2774 (*N* = 1).

**Figure 2:**
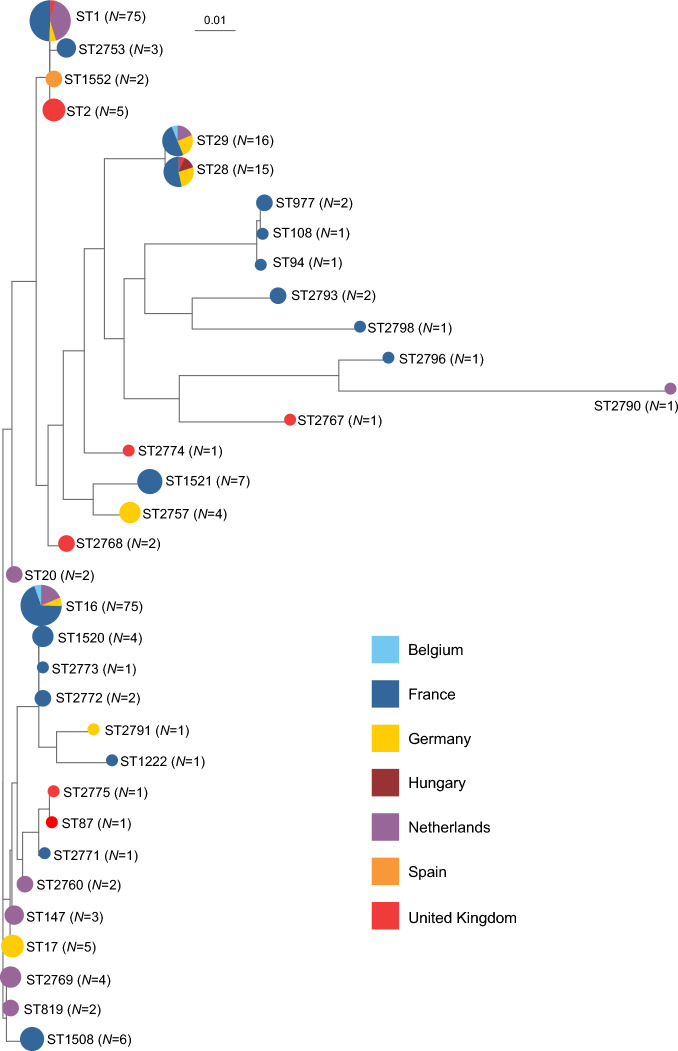
**Inferred genetic relationships between *S. suis* isolates recovered from diseased pigs in seven European countries based on the seven genes used in the multilocus sequence typing scheme (MLST).** A maximum likelihood phylogenetic tree was constructed by concatenating the sequences of genes (*aroA*, *cpn60*, *dpr*, *gki*, *mutS*, *recA*, and *thrA*) used in the *S. suis* MLST scheme. Each sequence type (ST) is plotted with the number of isolates (*N*) indicated beside each ST. Colored circles represent the countries of origin for the isolates—Belgium, France, Germany, Hungary, the Netherlands, Spain, and the United Kingdom, as per the key, providing a visual representation of the geographic distribution of genetic variants. Notably, ST1 and ST16 isolates were the most prevalent, but we identified a total of 34 STs, including 16 novel STs.

Serotype 7 isolates (*N* = 17) belonged to two genetically distantly related STs, ST29 (*N* = 16), and novel ST2771 (*N* = 1). Serotypes other than 7 were not identified in these two STs. Most serotype 1 isolates were ST1 (*N* = 23), while two others were ST1552, an ST1 derivative (*N* = 2). While the major STs were found in multiple countries, several other STs were country specific (Figure 2). The untypable isolates from Hungary belonged to ST28, while the two French and the Dutch untypable isolates belonged to novel ST2796, ST2798, an ST2790, respectively.

### Comprehensive phylogenetic analysis of European *S. suis* isolates reveals complex genetic diversity

#### Core-genome phylogenies and reference genomes

**Figure 3:**
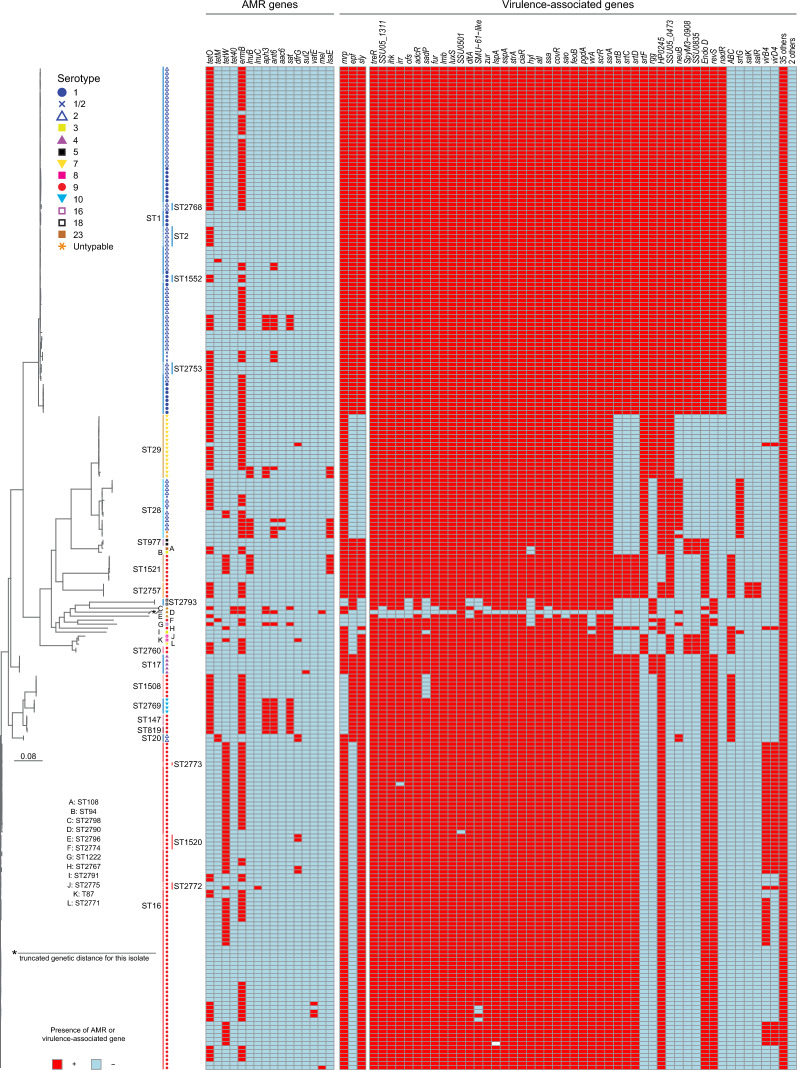
**Phylogenetic relationships based on core-genome single-nucleotide polymorphisms (SNPs), and genomic traits of the 251 *S. suis*** **isolates included in this investigation.** The left panel presents a maximum-likelihood phylogenetic tree constructed using 8558 non-redundant core-genome SNP loci identified among the isolates in the study collection relative to the genome sequence of the ST1 serotype 2 reference strain P1/7. The tree, delineating several distinct clades, highlights the genetic diversity among the isolates. For reference, the serotype of each isolate, as well as the genotypes determined by MLST, are annotated along the tree, illustrating their association with specific genomic clades. The right panel depicts the presence (in purple) or absence (in light blue) of antimicrobial resistance (AMR) genes, as well as virulence-associated genes (VAGs), as determined by interrogating the whole-genome sequences of each isolate. The varying distribution of AMR and VAGs across different clades suggests patterns of acquisition and retention of these elements.

We next expanded our genetic exploration and constructed core genome-based phylogenies, thus capturing a more comprehensive extent of genomic diversity beyond the seven housekeeping genes used in the *S. suis* MLST scheme. To identify SNPs, we used the genome sequence of strain P1/7 as the reference genome. P1/7 is a well-documented ST1 serotype 2 strain extensively used in *S. suis* research [52]. The alignment of the 251 isolates against this reference yielded a dataset of 8,557 non-redundant core-genome SNPs, providing high-resolution insights into the genetic diversity of the isolates (Figure 3). Further comparative analysis using the genome sequence of the ST16 serotype 9 strain GD-0088 [53], confirmed the tree topology, and demonstrated no significant deviation of the phylogenetic conclusions (Additional file 4). Inspection of the core-genome phylogenies revealed a very good agreement between genomic clades and MLST genotypes. In addition to several numerically minor clades, there were two major clades of isolates, one comprising ST1 strains of serotypes 1, 2, and 1/2, and one comprising ST16 serotype 9 strains. However, in transitioning from traditional MLST to more comprehensive core-genome SNP phylogenies, we observed a significant enhancement in the resolution of genetic relationships among the isolates and strong signatures of geographic diversification.

#### Geographic signatures of subclade diversity within ST1 S. suis isolates

Core genome phylogenies identified multiple subclades of ST1 serotype 2 isolates. With one exception, ST1 serotype 2 isolates from France, including the genetically closely related novel ST2753, did not cluster with serotype 2 strains from the Netherlands, while isolates from novel ST2768 were found only in the United Kingdom (Additional file 5). ST1 serotype 1/2 isolates were found in a single discrete subclade more closely related genetically to strains of serotype 2 than to those of serotype 1. These serotype 1/2 organisms were all from Germany (Additional file 5). The ST1 serotype 1 isolates clustered in four distinct, but relatively genetically close subclades, and presented a strong geographical signal of diversification. For example, one subclade included strains from the Netherlands as well as one German isolate, while 2 other subclades only had isolates from France. The fourth ST1 subclade included organisms from the United Kingdom, as well as two closely related ST1552 isolates from Spain (Additional file 5). Notably, we did not detect any intermixing of serotypes among the different subclades, which may be an indication that capsular switching between serotypes 2, 1 and 1/2 isolates does not occur frequently among ST1 isolates.

#### Subclade diversity in ST28 isolates

In agreement with previous reports from North America, where ST28 organisms are more commonly found and diversity within ST28 isolates has been described [54], we observed multiple subclades among European ST28 *S. suis* isolates (Additional file 6). The majority of the ST28 isolates were of serotype 2 (*N* = 11). The remaining 2 isolates were of serotype 1/2, were genetically distant between themselves, and originated from France and the United Kingdom. The French isolate was genetically more closely related to isolates of serotype 2 from France, while the one from the United Kingdom was closer to, although relatively distantly related from, ST28 serotype 2 isolates from Germany (Additional file 6). We also found that the 2 ST28 untypable isolates from Hungary clustered within a subclade of serotype 2 ST28 organisms from France. As described above, the Hungarian isolates presented a truncated *cps* locus whose remnants suggest they may have originated from a serotype 2 ancestor.

#### Unexpected high genetic diversity among serotype 9 isolates and potential for adaptive advantages of a Cps9 capsule

ST16 isolates, as well as isolates of closely related derivatives ST1520, ST2773 and ST2772 were all of serotype 9. We identified among them 5 subclades comprising isolates from France, one of which also had organisms recovered in the Netherlands (Figure 3 and Additional file 7). Another subclade was formed solely by German isolates, although they originated from the same farm, while ST16 serotype 9 organisms from Belgium were closely related to two other subclades comprising Dutch isolates. Interestingly, the ST16 serotype 9 organisms all had a classical VAG profile of *mrp* + , *epf*-, *sly*+ (Figure 3 and the next section), and relatively good conservation among the extended panel of 84 additional VAGs, but there were differences among the subclades for the presence of genes *virB4* and *virD4*, which have been connected to a *S. suis* type IV-like secretion system [55].

**Figure 4:**
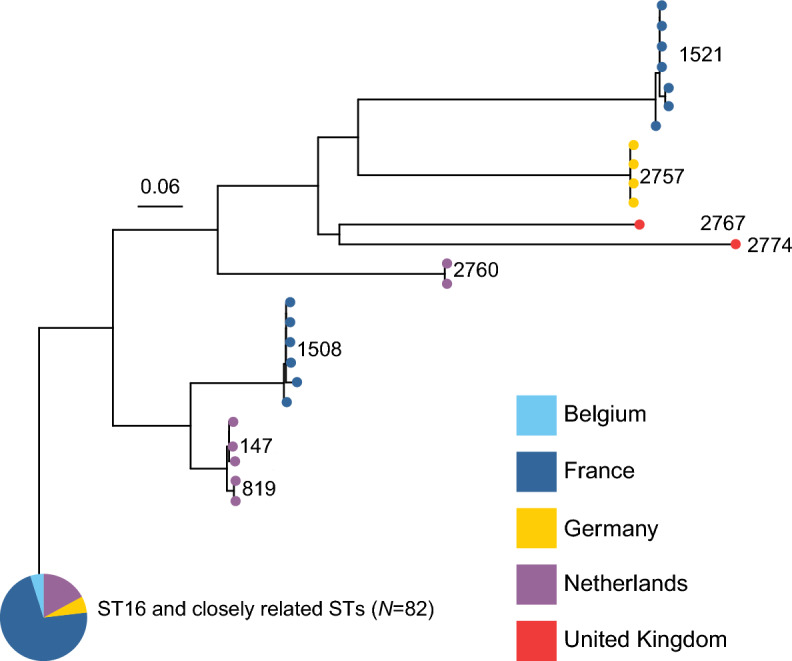
**Inferred genetic relationships among 108 serotype 9 *S. suis*** **strains investigated in this study based on core-genome analysis.** The maximum likelihood phylogenetic tree was constructed based on 15 934 non-redundant core-genome SNP loci identified relative to the genome sequence of the ST16 serotype 9 reference strain GD-0088. The tree reveals that serotype 9 isolates are distributed across several distinct genomic clades, including four newly discovered genotypes, highlighting complex evolutionary dynamics. These patterns suggest that the *cps9* Cps9 capsule may confer adaptive capabilities, although further research is needed to confirm such speculation. The tree is color-coded to indicate the country of origin for each strain, showcasing the geographical distribution across Belgium, France, Germany, the Netherlands, and the United Kingdom. The clade comprising ST16 organisms along with closely related STs 1520, 2772, and 2773 has been collapsed for simplicity, focusing the analysis on broader genomic diversity within serotype 9.

In addition to ST16 and its closely related derivatives (ST1520, ST2773 and ST2772), we observed that serotype 9 *S. suis* isolates also belonged to several other genetically distinct clades (Figure 4). These included ST1508 organisms from France, ST147 and ST819 isolates from the Netherlands, and ST1521 strains from France. Additionally, we identified that isolates belonging to previously undescribed genomic backgrounds, namely novel ST2757 from Germany, novel STs 2767 and 2774 from the United Kingdom, and novel ST2760 from the Netherlands all possessed a *cps9* locus. These four novel genomic backgrounds are genetically relatively distant from each other and from all previously described serotype 9 genomic backgrounds (Figure 4). This diversity in genomic backgrounds, compared to serotypes 2 and 1, might indicate a more frequent occurrence of capsular exchanges in some genomic backgrounds and a potential selective advantage for a serotype 9 capsule. However, other *cps* loci may also be exchanged in these potentially more recombination-permissive genomic backgrounds, as evidenced by serotype 10 isolates of the novel ST2769, which clustered with several non-ST16 serotype 9 organisms.

#### Other genetic backgrounds in S. suis isolates

ST29 organisms were all of serotype 7, and they separated into four main subclades (Additional file 8), with each subclade representing geographical diversification (organisms were from Belgium, France, Germany and the Netherlands, respectively). Other genetically highly similar organisms were ST17 serotype 4 isolates. Interestingly, the non-ST28 untypable isolates were all singletons, and genetically distantly related to all other groups of isolates based on core genome-based phylogenies, although one of these untypables isolates, the ST2798 from France, appeared to have shared a common ancestor with ST2793 serotype 18 isolates recovered in the same country (Figure 3).

### Distribution and diversity of virulence-associated genes in *S. suis* isolates

#### Screening for classical and for an extended panel of virulence associated genes (VAGs)

We interrogated the genome sequences for the presence of the 3 classical *S. suis* VAGs (genes *mrp*, *epf*, and *sly*), and found that they were present in 92.0%, 50.6%, and 84.5% of the isolates, respectively (Additional file 9). Only 3 isolates did not possess any of the 3 classical VAG genes, and 98.8% of all isolates (*N* = 248) contained at least one*,* with 35, 104, and 109 isolates possessing 1, 2, or all 3 classical VAGs, respectively. We identified 7 different classical VAG profiles in our collection. In addition to the abovementioned *mrp* + *, epf* + *, sly* + profile, with 109 isolates, we found isolates with *mrp* + *, epf*-*, sly* + (*N* = 88), *mrp* + *, epf*-*, sly*- (*N* = 33), and *mrp*-*, epf* + *, sly* + (*N* = 15). The other 3 classical VAG profiles were *mrp*-, *epf*-, *sly*- (*N* = 3), *mrp*-, *epf* + , *sly*- (*N* = 2), and *mrp* + , *epf* + , *sly*- (*N* = 1) (Additional file 9).

We next screened the genomes for the presence of 84 additional VAGs and found that 82 of them were present in at least one isolate (Additional file 10). Furthermore, the median amount of VAGs found in strains within our collection was 77. There were 35 unique VAGs present in all 251 isolates (Additional file 10). On the other hand, we did not identify among our isolates genes *nisK* and *nisR*, encoding a two-component signal-transduction system (TCS) first reported in Chinese *S. suis* ST7 serotype 2 strain SC84 (RefSeq NC_012924) and often found among ST7 serotype 2 isolates from that country [56, 57]. This TCS has been associated with human rather than with pig *S. suis* isolates [40]. A set of 26 PZVFs has been described and associated with human infections [40]. Of these, nine were included in our initial subset of 84 virulence-associated genes (VAGs) due to their broader relevance to virulence across different host species and were thus screened as part of our primary analysis. The remaining 17 PZVFs, which might be more specifically associated with zoonotic transmission, were screened separately. Data for these additional 17 PZVFs is presented in Additional file 11.

#### Serotype and sequence type-specific distribution of virulence associated genes

Isolates of serotypes 1, 1/2, and 2 clustering in the phylogenetic group comprising ST1 and derivatives such as ST2, closely related ST1552, ST2753, and ST2768, had the highest number of VAGs (79 out of 87 including the classical VAGs, Figure 3, and Additional files 9 and 10). In more detail, all isolates were: *mrp* + , *epf* + , *sly* + , and only lacked, in addition to *nisKR*, the genes *virB4*, *virD4*, *salK*, *salR*, *srtG*, and *SSUST3_0534/ABC*. Furthermore, isolates of this group exhibited the least VAG content diversity compared to other major phylogenetic groups.

In contrast to the ST1 cluster, the cluster containing ST28 isolates (of serotypes 1/2 and 2, as well as 2 untypable isolates), and the cluster of ST29 of serotype 7 were characterized by a classical VAG profile of *mrp* + , *epf*-, *sly*-, and by fewer additional VAGs relative to the ST1 group. The extended screening identified that within each of these groups, isolates had highly consistent patterns of VAG presence. However, there were differences in extended VAG content between ST28 and ST29 isolates (*N* = 5; *rgg*, *virB4*, *virD4*, *neuB*, and *srtG*) (Additional files 9 and 10). The divergent ST2790 untypable isolate NSUI00477 from the Netherlands had the lowest amount of VAGs (41 out of 87, 47.1%).

Among the 108 serotype 9 isolates in our collection, 4 different classical VAG profiles were found. Isolates of serotype 9 belonging to ST16, ST1520, ST2760 and ST2767 (*N* = 85, 78.7%) had an *mrp* + , *epf*-, *sly* + profile. Serotype 9 isolates belonging to ST147, ST819, and ST1508 (*N* = 11, 10.2%) had an *mrp*-, *epf* + , *sly* + profile, while ST1521 and novel ST2757 serotype 9 isolates (*N* = 11, 10.2%) possessed all 3 classical VAGs *mrp* + , *epf* + , *sly* + . One serotype 9 isolate, belonging to novel ST2774, did not possess any of the 3 classical VAGs. Out of all 87 putative VAGs, 58 VAGs were present in all serotype 9 strains, while 5 VAGs (*neuB*, *nadR*, *srtG*, *nisK*, and *nisR*) were absent from all serotype 9 isolates (Additional files 9 and 10). The extended VAG content among the 108 serotype 9 strains varied significantly, with up to 11 gene differences. However, there was less variation among the serotype 9 isolates belonging to ST16 and close derivatives, which differed in VAG content by 3 or fewer VAGs. In this subgroup, 68 out of 87 VAGs were present in all strains and 14 out of 87 VAGs were consistently absent (Additional files 9 and 10).

### Prevalence and distribution of antimicrobial resistance genes in European *S. suis*

We identified a total of 16 different AMR genes potentially conferring AMR resistance in our *S. suis* isolate collection (Additional file 1 and Figure 3). 214 strains (85.3%) possessed at least 1 AMR gene, whereas the remaining 37 strains (14.7%) did not possess any. Most isolates (*N* = 129, 51.4%) had 2 AMR genes, while 49 (19.5%) isolates had only 1 AMR gene. Isolates with 3 or more AMR genes represented 14.3% (*N* = 36) of the collection. The highest number of AMR genes found in a single isolate was 6 (*tetO*, *ermB*, *aph3*, *ant6*, *lnuB*, *lsaE*) in an ST2771 serotype 7 organism recovered in Belgium.

Two AMR genes, *tetO* (*N* = 124, 49.4%) and *ermB* (*N* = 163, 64.9%), encoding resistance to tetracycline, and to streptogramin (A & B), macrolide and lincosamide antimicrobial drug classes, respectively, were the most frequently observed. There were 108 strains (43.0%) that possessed both genes. Gene *tetW* was found in 61 isolates (24.3%). In addition to *tetO* and *tetW*, we also observed genes *tet40* and *tetM* in several isolates (Additional file 1). In total, 190 isolates (75.7%) possessed at least one gene potentially conferring resistance to tetracycline. Eight AMR genes (*lnuC*, *tet40*, *mel*, *dfrG*, *aac6*, *vatE*, *sul2*, *tetM*) were present in less than 5% of isolates in the collection (Additional 1).

Among the 108 serotype 9 isolates, 13 (12.0%) did not possess any AMR genes (Table 1). A total of 58 serotype 9 isolates (53.7%) had gene *tetW*, accounting for 95.1% of the total 61 isolates in our collection having this gene. Additionally, of the 163 isolates carrying the *ermB* gene, 66 were serotype 9 isolates, which represents 40.5% of the isolates with this gene and 61.1% of all serotype 9 isolates tested. Interestingly, no serotype 9 isolates possessed genes *tet40*, *aac6*, and *sul2*. In the subgroup of ST16 serotype 9, only 7 out of the total 16 AMR genes identified in the full collection were detected. In addition to the previously mentioned three genes, genes *sat*, *ant6*, *lnuB*, *lsaE*, and *tetM* were absent from the genomes of ST16 serotype 9 strains. Thus, compared to the broader group of serotype 9 isolates, ST16 serotype 9 isolates had lower AMR gene diversity.

**Table 1:** **Antimicrobial resistance (AMR) gene content among the *Streptococcus suis* isolates used in this study**

Serotype	Number of isolates	AMR genes															
		*tetO*	*tetM*	*tetW*	*tet40*	*ermB*	*lnuB*	*lnuC*	*aph3*	*ant6*	*aac6*	*sat*	*dfrG*	*sul2*	*vatE*	*mel*	*lsaE*
1	25	19	0	0	0	19	0	0	0	0	0	0	0	0	0	0	0
1/2	5	5	0	0	0	4	0	0	0	3	0	0	0	0	0	0	0
2	72	47	3	2	0	47	3	0	4	8	2	4	2	0	0	0	3
3	2	1	0	0	0	1	0	0	0	0	0	0	0	0	0	0	0
4	5	0	0	0	0	0	0	0	0	0	0	0	0	1	0	0	0
5	2	0	0	0	0	0	0	0	0	0	0	0	0	0	0	0	0
7	17	14	0	0	0	15	3	0	3	1	0	0	1	0	0	0	3
8	2	0	0	1	0	1	0	0	0	0	0	0	1	0	0	0	0
9	108	30	1	58	0	66	5	1	5	5	0	5	4	0	3	1	5
10	4	4	0	0	0	4	0	0	4	4	0	4	0	0	0	0	0
16	1	1	0	0	0	1	0	0	1	1	0	1	0	0	0	0	0
18	2	0	0	0	0	0	0	0	0	0	0	0	0	0	0	0	0
23	1	1	0	0	0	1	0	0	0	0	0	0	0	0	0	0	0
UT^a^	5	2	0	0	2	4	2	0	2	3	0	1	0	0	0	1	2
Total	251	124	4	61	2	163	13	1	19	25	2	15	8	1	3	2	13

^a^UT: Untypable isolate.

Among the 72 serotype 2 isolates, 8 did not possess any AMR genes (Table 1). However, as a population the serotype 2 organisms had 11 out of the 16 AMR genes detected in the full collection (genes *tet40*, *lnuC*, *vatE*, *mel*, and *sul2* were absent from all isolates in this group, Additional file 1). Notably, 47 serotype 2 isolates possessed gene *tetO* (65.3% of the serotype 2 isolates, and 37.9% of all isolates with this gene). The diversity of AMR gene presence in the group of ST1 serotype 2 organisms was lower compared to all serotype 2 isolates. Indeed, only 6 of the 16 total AMR genes were detected (i.e., in addition to the three above mentioned AMR genes, we did not detect genes *tetW*, *lnuB*, *lsaE*, *dfrG*, and *aac6*). These results seem to align with previous reports describing a paucity of AMR-carrying mobile genetic elements among ST1 *S. suis* in comparison to serotype 2 strains of other genomic backgrounds [32, 58–60]. Interestingly, among the 25 ST1 serotype 1 isolates, we only identified 2 AMR genes (*tetO* and *ermB*), but 19 serotype 1 isolates (76.0%) carried both *tetO* and *ermB* genes. All serotype 7 isolates (*N* = 17) carried at least 2 AMR genes: 13 isolates possessed genes *tetO* and *ermB*, 2 isolates possessed genes *aph3, lnuB* and *lsaE*, 1 isolate possessed genes *ermB* and *dfrG*, and 1 isolate possessed 6 AMR genes: *tetO*, *ermB*, *aph3*, *ant6*, *lnuB*, and *lsaE*. Isolates of serotypes 5 and 18 did not possess any AMR genes, although we note that these serotypes were represented by 4 isolates only, accounting for only 1.6% of the collection.

## Discussion

### Genomic insights and pathogen diversity

We report here considerable diversity in serotypes and genomic backgrounds among *S. suis* strains recovered from diseased pigs in Europe. The wealth of the data permitted us to identify several novel derivatives of previously reported STs, as well as new genomic backgrounds. Our findings underscore the superior discriminatory power of genomics for detecting subtle genetic variations that are important for tracking pathogen evolution and epidemiology [12, 13, 61, 62].

Core-genome single SNP analysis was key in highlighting nuanced interactions within phylogenetic clusters, revealing a complexity in genetic interactions not previously fully appreciated. For example, the identification of these novel STs and genomic backgrounds among our isolate collection reinforces previous reports describing the ongoing genetic evolution within the *S. suis* population [63], and suggests a dynamic *S. suis* genetic landscape where new variants continually emerge, possibly as a response to selective pressures such as host immune responses (due to natural infections or autogenous vaccines) or antibiotic treatments. This genetic fluidity underscores the adaptability of *S. suis*, reinforcing the need for continuous surveillance using advanced molecular tools [15–17, 62]. Furthermore, we also clearly show regional differences in genetic evolution. Monitoring geographical variation in STs and serotypes can inform targeted health interventions in the swine industry and enhance understanding of *S. suis* transmission dynamics. Such monitoring also supports broader public health efforts, defined here to include the management of zoonotic risks and the health of animal populations, which are integral to community health strategies [11, 64, 65].

One of our findings was a potential for genetic “promiscuity” among serotype 9 isolates, which were represented in several genetically distantly related genomic backgrounds. This may imply a more frequent occurrence of capsular switching and genetic recombination and a potential capacity of serotype 9 capsules to provide an adaptive mechanism that may confer survival advantages under various environmental pressures, such as immune evasion or antimicrobial exposure [11, 16, 66]. This finding was in contrast with our results for serotypes 1, 2, and 1/2, which generally exhibited more genetic stability and uniformity, indicating a potentially more conserved evolutionary path. However, the recent emergence of serotype 2 ST20 also illustrates that evolutionary diversification, while perhaps less pronounced, does occur in these typically more stable serotypes. It has been suggested in previous work that ST20 organisms may have originated from ST16 strains [67]. Our data confirms that ST20 and ST16 organisms are relatively closely related genetically. However, because ST20 isolates have patterns of SNP distribution similar to those observed for ST147 and ST819 serotype 9 isolates when compared against an ST16 genome (Additional file 12), our data also suggest that it is unlikely that recombinant ST20 serotype 2 organisms have originated directly from an ST16 ancestor by a single recombination event involving capsular switching. This complex adaptive landscape may reflect the varying evolutionary pressures and strategies employed by different serotypes within the species [12, 13, 66]. It is also important to mention that the diversity we discovered within serotype 9 poses challenges for molecular typing and epidemiological tracking. Traditional serotyping and some molecular methods cannot fully capture the genetic diversity of *S. suis*, especially when capsular gene swapping may be involved. Advanced genomic tools can provide the resolution necessary to accurately characterize these dynamic genetic changes, offering insights into the evolutionary pressures shaping the *S. suis* development [62, 68]. Our study also identified a subset of *S. suis* isolates that were untypable; some appear to have lost the ability to express a capsule, while others may represent novel capsular types. Since these isolates were recovered from diseased animals, further characterization of these untypable organisms might be clinically significant and important for enhancing diagnostics.

The *S. suis* genomic diversity revealed through our study further highlights the need for innovative vaccine strategies for control of this organism. Traditional vaccines targeting specific serotypes may not suffice due to the emergence of genetically diverse strains and the apparent notable adaptability of serotypes like serotype 9 [69, 70]. Thus, our findings advocate for next-generation vaccines that target more conserved elements across the species or are adaptable to evolving genetic variants. Additionally, leveraging genomic insights can enhance autovaccine formulations by ensuring they are tailored to combat the most prevalent and virulent strains, providing a targeted response to local disease challenges [3, 7, 19]. By integrating genomic data into vaccine design, it may be possible to improve the efficacy of autovaccines [71], potentially reducing the incidence of *S. suis* outbreaks and mitigating their impact on swine health and the associated economic consequences.

### Virulence factor analysis

VAG content analysis can provide insights into the capacity of an isolate to cause disease [72]. The classical VAGs (*epf*, *mrp*, and *sly*) are factors with proven or potential roles in the pathogenesis of the *S. suis* infection: EF, encoded by *epf*, has been postulated to enhance the bacterial survival in host tissues, MRP has been associated to immune evasion, and suilysin shown to contribute to septicemia and meningitis by lysing host cells [34, 35]. The consistent profiles of these genes across different serotypes and STs underscores their role as markers of virulence in *S. suis* [1]. In addition, our study also revealed a broad distribution of additional putative VAGs that might influence the *S. suis* fitness and virulence in more nuanced ways. For instance, genes putatively involved in adhesion, toxin production, and immune modulation were variably present across isolates, further stressing that different strains of *S. suis* might be equipped with unique arsenals for interaction and colonization of the host [36]. This variation could explain reports on differential pathogenicity among strains and their varying ability to cause outbreaks of differing severity in swine populations [34, 35]. Moreover, the serotype-specific variability in VAG profiles highlighted in our study indicates that virulence traits may be influenced by the genetic context of the serotype. For example, the genetic “promiscuity” of serotype 9, as well as the potential for capsular switching in *S. suis* might lead to combinations of VAGs that confer advantages in particular environmental or host contexts, potentially complicating vaccine development and therapeutic interventions aimed at controlling infections [1, 13]. We extended our analysis to examine the presence of 26 additional PZVFs across isolates in our collection. We found that the genes encoding all 26 PZVFs were variably present across the isolates but with consistent patterns across different genotypes suggesting PZVFs could serve as markers for zoonotic pathogenesis and virulence in *S. suis* isolates, as previously suggested [40].

Our findings also stress the importance of using advanced genomic tools to map the VAG landscape comprehensively. Traditional phenotypic assays and limited gene panels may not capture the full spectrum of virulence factors, especially those newly recognized or less studied. Genomics enables the identification of novel VAGs and provides insights into their co-occurrence patterns, which are crucial for understanding how combinations of virulence factors may interact to enhance pathogenicity [72, 73].

### AMR profiles and public health implications

The widespread resistance to tetracyclines, macrolides, and lincosamides across various serotypes and sequence types shown in our study is in line with previous reports [21, 26–28, 31, 32, 58, 59, 65, 74, 75]. While these antimicrobials are not used for the treatment of *S. suis*, they are routinely used to treat other bacterial infections affecting swine, such as respiratory diseases caused by *Mycoplasma hyopneumoniae* and *Actinobacillus pleuropneumoniae*, as well as gastrointestinal disorders linked to *Lawsonia intracellularis* [76, 77]. The routine use of these drugs could potentially influence the transmission of resistance traits among bacterial populations. This underlines the need for ongoing surveillance and responsible management of antibiotic usage to mitigate broader impacts on antimicrobial resistance dissemination. Our data show that there were not consistent correlations between AMR profiles and specific genetic backgrounds, suggesting that resistance traits disseminate across diverse *S. suis* populations via horizontal gene transfer. The transmission of *S. suis* from swine to humans, primarily affecting those in close contact with pigs or pork products, can lead to serious infections, such as meningitis and septicemia [1, 7]. The overlap in antimicrobial use between humans and swine amplifies this risk, as resistance mechanisms developed in swine can readily transfer to human pathogens, a scenario of significant concern under the One Health approach [26]. While this may not readily apply to Europe, the implications for public health are particularly critical in regions of the world where regulatory oversight of antimicrobial use in agriculture is less stringent. In such settings, the risk of AMR gene exchange between *S. suis* and other human pathogens increases, and could potentially lead to outbreaks of drug-resistant infections that are difficult to control [28, 29]. This genetic mobility necessitates vigilant monitoring and regulation of antibiotic use in swine production to prevent further development and spread of resistance [27, 31].

### Methodological considerations and study limitations

We used a convenience sample of 251 isolates collected across seven European countries, providing a comprehensive overview of *S. suis* diversity in terms of serotypes, AMR profiles, and virulence factors. The strategic selection of these isolates, many of which were either used or considered for inclusion in autovaccine formulations, ensures that our findings are directly relevant and reflective of the strains that are most significant for disease management in commercial swine production. This aspect of our sampling strategy lends a practical utility to our dataset, as it mirrors the types of strains that pose the greatest concern in field conditions [1]. The geographical and temporal breadth of the sample collection, spanning from 2012 to 2020, captures variability in *S. suis* populations and the potential impact of varying farm management practices and antibiotic usage patterns [11, 31].

While the convenience sample offers practical insights, it may also introduce biases that could affect the generalizability of our findings [78]. However, the consistency and similarity observed across diverse isolates suggest that our results offer a credible reflection of the wider *S. suis* population dynamics within European swine herds. To further validate and extend our observations, future studies could employ random sampling and expand the geographic and host species scope to include more countries and potentially other susceptible European animal populations such as wild boars, which have been found to sometimes carry *S. suis* strains of similar genotypes and serotypes as the ones described here [58, 79, 80]. Such studies would enrich our understanding of *S. suis* epidemiology and control strategies, reinforcing the relevance of our current findings and facilitating the development of more targeted interventions.

Our study has revealed extensive genomic diversity in *S. suis* across Europe, highlighting the adaptability and evolutionary dynamics of this pathogen. Key findings include the notable genetic variability within serotype 9 and its implications for disease management and vaccine development. This underscores the importance of employing advanced genomic methodologies for ongoing surveillance and the creation of adaptable vaccine strategies that can respond to this diversity. Emphasizing a One Health approach, we advocate for integrated strategies that consider the interconnected health impacts across human, animal, and environmental spheres to effectively manage and mitigate *S. suis* infections. This approach is essential not only for enhancing animal health but also for protecting public health, given the zoonotic potential of *S. suis*.

## Supplementary Information

**Additional file 1. 251 *Streptococcus suis *isolates used in this study.** This file contains a table of 251 *Streptococcus suis *strains used for analyses in this study, including information regarding country of isolation, genome information, serotype and STs, presences of AMR genes, and Biosample accession numbers.

**Additional file 2. Comparative architecture of *cps *loci in untypable** ***Streptococcus suis *isolates.** Illustrated is the genetic organization of the *cps* loci of the five *S. suis* untypable isolates, including a comparison to appropriate reference *cps* loci sequences. Percentage identity to reference sequences is indicated. **A)** ST28 isolates NSUI00643 and NSUI00644, both recovered in Hungary, possessed some but lacked several key *cps* genes found in the reference ST28 serotype 2 strain NSUI002 (GenBank accession number CP011419.1). **B)** ST2796 isolate NSUI00607, recovered in France, possessed some but lacked several key *cps* genes found in serotype 31 strain 92-4172 (GenBank accession number AB737835.1). **C)** ST2790 isolate NSUI00477, recovered in the Netherlands, possessed some but lacked several key *cps* genes found in reference serotype 1 strain 5428 (GenBank accession number JF273644.1). **D**) ST2798 isolate NSUI00633, recovered in France, possessed some but lacked several key *cps* genes found in reference serotype 28 strain 89-590 (GenBank accession number AB737832.1).

**Additional file 3. STs identified among the *Streptococcus suis *isolates used in this study.** This file contains a table of STs distribution of 251 *S. suis *isolates used in this study along with their respective housekeeping gene allele number.

**Additional file 4.** **Phylogenetic relationships based on core-genome single-nucleotide polymorphisms (SNPs) and genomic traits of the 251 *Streptococcus*** ***suis* isolates using a different reference genome.** This figure presents a maximum-likelihood phylogenetic tree (left panel), constructed using 8,611 non-redundant core-genome SNP loci identified relative to the genome sequence of the ST16 serotype 9 reference strain GD-0088. This analysis confirms the findings depicted in Figure 3, using a different reference to provide comparative insights. The tree highlights several distinct clades, emphasizing the genetic diversity among the isolates. For reference, the serotype of each isolate, along with the genotypes determined by multilocus sequence typing (MLST), are annotated along the tree, showing their association with specific genomic clades. The right panel depicts the presence (in purple) or absence (in light blue) of antimicrobial resistance (AMR) genes and virulence-associated genes (VAGs), as determined from the whole-genome sequences of each isolate. “UT” denotes an untypable isolate.

**Additional file 5.  Inferred genetic relationships based on core-genome analysis among the 82 *Streptococcus suis*** **isolates investigated in this study belonging to sequence type (ST) 1 and closely related STs.** The maximum likelihood phylogenetic tree was constructed from 6,272 non-redundant core-genome SNP loci identified relative to the genome sequence of the ST1 serotype 2 reference strain P1/7. Each isolate is uniquely identified (e.g., NSUI00470) and annotated with its serotype and ST. The tree includes isolates from France, Germany, the Netherlands, Spain, and the United Kingdom, with each country represented by a specific color. This illustrates the geographic distribution of the strains and highlights that patterns of strain diversification have a strong geographic signature.

**Additional file 6. Inferred genetic relationships based on core-genome analysis among the 15 *Streptococcus suis*** **isolates belonging to sequence type 28 investigated in this study.** The maximum likelihood phylogenetic tree was constructed from 8906 non-redundant core-genome SNP loci identified relative to the genome sequence of the ST28 serotype 2 reference strain NSUI002 (GenBank Accession number CP011419.1). Each isolate is uniquely identified (e.g., NSUI00524) and annotated with its 24 serotype and ST. The tree includes isolates from France, Germany, Hungary, and the United Kingdom, with each country represented by a specific color. “UT” denotes an untypable.

**Additional file 7. Inferred genetic relationships based on core-genome analysis among the 75 *Streptococcus suis*** **isolates belonging to sequence type 16 investigated in this study.** The maximum likelihood phylogenetic tree was constructed from 8906 non-redundant core-genome SNP loci identified relative to the genome sequence of the ST16 serotype 9 reference strain GD-0088. Each isolate is uniquely identified (e.g., NSUI00524) and annotated with its serotype and ST. The tree includes isolates from Belgium, France, Germany, and the Netherlands, with each country represented by a specific color.

**Additional file 8. Inferred genetic relationships based on core-genome analysis among the 16 *S. suis*** **isolates belonging to sequence type 29 investigated in this study. **The maximum likelihood phylogenetic tree was constructed from 645 non-redundant core-genome SNP loci identified relative to the genome sequence of the ST29 serotype 7 reference strain 13-00283-02 (GenBank Accession number NZ_CP058741.1). Each isolate is uniquely identified (e.g., NSUI00528) and annotated with its serotype and ST. The tree includes isolates from France, Germany, Hungary, and the United Kingdom, with each country represented by a specific color.

**Additional file 9. Distribution of classical virulence-associated genes (VAGs) across *Streptococcus suis *serotypes.** This file contains a table of the association of *S. suis *serotypes and the identified classical virulence-associated gene (*mrp*, *epf*, *sly*) profiles.

**Additional file 10. Detection of virulence-associated genes (VAGs) in the *Streptococcus suis *isolates used in this study.** This file contains a list of 80+ VAGs screened in each *S. suis *strains in this study, including differential presences in different sequence types.

**Additional file 11. Detection   of 17 additional putative zoonotic virulence factors (PZVFs) among 251** ***Streptococcus suis*** **isolates.** This figure presents a maximum-likelihood phylogenetic tree (left panel), constructed using 8,558 non-redundant core-genome SNP loci identified relative to the genome sequence of the ST1 serotype 2 reference strain P1/7. The right panel depicts general strain information (serotype, clonal complex, country; 3 left-most columns), and PZVFs presence (black) or absence (white) as determined from the whole-genome sequences of each isolate. Isolates of serotypes 1, 2 and 1/2 belonging to CC1 had more PZVFs than other serotypes.

**Additional file 12. Genomic synteny of *Streptococcus suis*** **ST20, ST147, and ST819 strains in comparison to ST16 serotype 9 reference strain GD-0088.** Coverage data (innermost circles) and single-nucleotide polymorphisms (SNPs, outermost circles) for strains NSUI00645 (ST20), NSUI00474 (ST147) and NSUI00682 (ST819) are plotted against the ST16 serotype 9 reference strain GD-0088. The similarity in SNP distribution patterns across several areas of the genome between the ST20, the ST147 and the ST819 isolates does not support the hypothesis that ST20 strains are derived directly from an ST16 organism. There were 13934, 16601, 16113 SNPs for strains NSUI00645, NSUI00474, and NSUI00682, respectively, relative to the reference strains. The position of the *cps9* locus in the reference genome is provided as a reference.

## Data Availability

Raw read data for *S. suis* isolates whose genomes were sequenced in this study have been deposited into the National Center for Biotechnology Information Sequence Read Archive under Bioproject accession number PRJNA1099673.
