# Supplementary material for: Molecular characterization of Streptococcus suis isolates recovered from diseased pigs in Europe
Source: Vet Res. 2024 Sep 27;55:117. doi: 10.1186/s13567-024-01366-y (PMC11429987; doi:10.1186/s13567-024-01366-y)
Supplement: Supplementary file 1 — Additional file 1. 251 Streptococcus suis isolates used in this study. This file contains a table of 251 Streptococcus suis strains used for analyses in this study, including information regarding country of isolation, genome information, serotype and STs, presences of AMR genes, and Biosample accession numbers. [file 13567_2024_1366_MOESM1_ESM.docx]

**Additional file 1. *Streptococcus suis* isolates used in this study.**

| Isolate Name | Country of Isolation | Genome Size^a^ | Number of Reads | Depth of Coverage^b^ | Serotype | | ST^c^ | Antimicrobial resistance gene | | | | | | | | | | | | | | | | Biosample^d^ | SRA ID^e^ |
| --- | --- | --- | --- | --- | --- | --- | --- | --- | --- | --- | --- | --- | --- | --- | --- | --- | --- | --- | --- | --- | --- | --- | --- | --- | --- |
|  |  |  |  |  | PCR | in silico |  | *tetO* | *tetM* | *tetW* | *tet40* | *ermB* | *lnuB* | *lnuC* | *aph3* | *ant6* | *aac6* | *sat* | *dfrG* | *sul2* | *vatE* | *mel* | *lsaE* |  |  |
| NSUI00402 | France | 2052555 | 550293 | 36.38 | 7 | 7 | 29 | + | - | - | - | + | - | - | - | - | - | - | - | - | - | - | - | SAMN40946562 | 40946562 |
| NSUI00441 | France | 2386513 | 654755 | 83.71 | 9 | 9 | 16 | - | - | + | - | + | - | - | - | - | - | - | - | - | - | - | - | SAMN40946563 | 40946563 |
| NSUI00443 | France | 2428308 | 172534 | 21.77 | 9 | 9 | 16 | - | - | + | - | + | - | - | - | - | - | - | - | - | - | - | - | SAMN40946564 | 40946564 |
| NSUI00445 | France | 2026784 | 163438 | 20.33 | 2 | 2 | 2753 | + | - | - | - | - | - | - | - | - | - | - | - | - | - | - | - | SAMN40946565 | 40946565 |
| NSUI00446 | France | 2301367 | 971999 | 82.13 | 9 | 9 | 2772 | - | - | + | - | - | - | + | - | - | - | - | - | - | - | - | - | SAMN40946566 | 40946566 |
| NSUI00447 | France | 2245464 | 838621 | 70.63 | 9 | 9 | 2772 | - | - | - | - | - | - | - | - | - | - | - | - | - | - | - | - | SAMN40946567 | 40946567 |
| NSUI00448 | France | 2353908 | 297253 | 25.59 | 9 | 9 | 16 | - | - | + | - | + | - | - | - | - | - | - | - | - | - | - | - | SAMN40946568 | 40946568 |
| NSUI00449 | France | 2378419 | 263057 | 23.75 | 9 | 9 | 16 | - | - | + | - | + | - | - | - | - | - | - | - | - | - | - | - | SAMN40946569 | 40946569 |
| NSUI00450 | France | 2397967 | 262268 | 33.15 | 9 | 9 | 16 | - | - | + | - | + | - | - | - | - | - | - | - | - | - | - | - | SAMN40946570 | 40946570 |
| NSUI00452 | France | 2438452 | 426032 | 52.42 | 9 | 9 | 16 | - | - | + | - | + | - | - | - | - | - | - | - | - | - | - | - | SAMN40946571 | 40946571 |
| NSUI00453 | France | 2369883 | 410742 | 35.27 | 9 | 9 | 16 | - | - | + | - | + | - | - | - | - | - | - | - | - | - | - | - | SAMN40946572 | 40946572 |
| NSUI00454 | France | 2094504 | 547448 | 61.24 | 9 | 9 | 16 | - | - | - | - | - | - | - | - | - | - | - | - | - | - | - | - | SAMN40946573 | 40946573 |
| NSUI00455 | France | 2103676 | 1428927 | 115.07 | 9 | 9 | 16 | - | - | - | - | - | - | - | - | - | - | - | - | - | - | - | - | SAMN40946574 | 40946574 |
| NSUI00456 | France | 2131546 | 1033574 | 80.95 | 2 | 2 | 28 | + | - | - | - | + | - | - | - | - | - | - | - | - | - | - | - | SAMN40946575 | 40946575 |
| NSUI00457 | France | 2136437 | 2016495 | 179.53 | 2 | 2 | 28 | + | - | - | - | + | - | - | - | - | - | - | - | - | - | - | - | SAMN40946576 | 40946576 |
| NSUI00458 | France | 2036530 | 520990 | 43.82 | 5 | 5 | 977 | - | - | - | - | - | - | - | - | - | - | - | - | - | - | - | - | SAMN40946577 | 40946577 |
| NSUI00459 | France | 2053097 | 1301319 | 101.37 | 5 | 5 | 977 | - | - | - | - | - | - | - | - | - | - | - | - | - | - | - | - | SAMN40946578 | 40946578 |
| NSUI00460 | France | 2048218 | 1003782 | 79.22 | 2 | 2 | 1 | + | - | - | - | + | - | - | + | + | - | + | - | - | - | - | - | SAMN40946579 | 40946579 |
| NSUI00462 | France | 2186231 | 1299990 | 109.52 | 9 | 9 | 16 | - | - | + | - | - | - | - | - | - | - | - | - | - | - | - | - | SAMN40946580 | 40946580 |
| NSUI00463 | France | 2182940 | 702588 | 62.72 | 9 | 9 | 16 | - | - | + | - | - | - | - | - | - | - | - | - | - | - | - | - | SAMN40946581 | 40946581 |
| NSUI00464 | Germany | 2064821 | 443380 | 39.42 | 1 | 1 | 1 | + | - | - | - | + | - | - | - | - | - | - | - | - | - | - | - | SAMN40946582 | 40946582 |
| NSUI00465 | Netherlands | 2077949 | 409658 | 33.26 | 1 | 1 | 1 | + | - | - | - | + | - | - | - | - | - | - | - | - | - | - | - | SAMN40946583 | 40946583 |
| NSUI00466 | Netherlands | 2070104 | 305245 | 24.98 | 1 | 1 | 1 | + | - | - | - | + | - | - | - | - | - | - | - | - | - | - | - | SAMN40946584 | 40946584 |
| NSUI00467 | Netherlands | 2082001 | 468363 | 44.79 | 1 | 1 | 1 | + | - | - | - | + | - | - | - | - | - | - | - | - | - | - | - | SAMN40946585 | 40946585 |
| NSUI00468 | Netherlands | 2083066 | 575147 | 51.09 | 1 | 1 | 1 | + | - | - | - | + | - | - | - | - | - | - | - | - | - | - | - | SAMN40946586 | 40946586 |
| NSUI00470 | Netherlands | 2082681 | 610020 | 55.12 | 1 | 1 | 1 | + | - | - | - | + | - | - | - | - | - | - | - | - | - | - | - | SAMN40946587 | 40946587 |
| NSUI00471 | Netherlands | 2080082 | 927006 | 82.57 | 1 | 1 | 1 | + | - | - | - | + | - | - | - | - | - | - | - | - | - | - | - | SAMN40946588 | 40946588 |
| NSUI00472 | Netherlands | 2080964 | 308411 | 26.27 | 1 | 1 | 1 | + | - | - | - | + | - | - | - | - | - | - | - | - | - | - | - | SAMN40946589 | 40946589 |
| NSUI00474 | Netherlands | 2172247 | 1112591 | 95.88 | 9 | 9 | 147 | + | - | - | - | + | - | - | + | + | - | + | - | - | - | - | - | SAMN40946590 | 40946590 |
| NSUI00475 | Netherlands | 2114431 | 307569 | 25.9 | 9 | 9 | 147 | + | - | - | - | + | - | - | + | + | - | + | - | - | - | - | - | SAMN40946591 | 40946591 |
| NSUI00476 | Netherlands | 2154482 | 312477 | 29 | 9 | 9 | 147 | + | - | - | - | + | - | - | + | + | - | + | - | - | - | - | - | SAMN40946592 | 40946592 |
| NSUI00477 | Netherlands | 2352563 | 1074323 | 75.27 | UT | UT | 2790 | - | - | - | + | + | - | - | + | + | - | - | - | - | - | + | - | SAMN40946593 | 40946593 |
| NSUI00478 | France | 2091440 | 809688 | 76.81 | 7 | 7 | 29 | + | - | - | - | + | - | - | - | - | - | - | - | - | - | - | - | SAMN40946594 | 40946594 |
| NSUI00480 | France | 1925320 | 175429 | 16.17 | 7 | 7 | 29 | - | - | - | - | - | + | - | + | - | - | - | - | - | - | - | + | SAMN40946595 | 40946595 |
| NSUI00481 | France | 2025551 | 991947 | 89.15 | 7 | 7 | 29 | - | - | - | - | - | + | - | + | - | - | - | - | - | - | - | + | SAMN40946596 | 40946596 |
| NSUI00482 | France | 2098539 | 497318 | 59.27 | 9 | 9 | 16 | - | - | - | - | - | - | - | - | - | - | - | - | - | - | - | - | SAMN40946597 | 40946597 |
| NSUI00483 | France | 2102805 | 1074350 | 118.33 | 9 | 9 | 16 | - | - | - | - | - | - | - | - | - | - | - | - | - | - | - | - | SAMN40946598 | 40946598 |
| NSUI00484 | Germany | 2140656 | 1190333 | 132.63 | 2 | 2 | 28 | + | - | - | - | - | - | - | - | - | - | - | - | - | - | - | - | SAMN40946599 | 40946599 |
| NSUI00485 | Germany | 2141859 | 1536863 | 174.45 | 2 | 2 | 28 | + | - | - | - | - | - | - | - | - | - | - | - | - | - | - | - | SAMN40946600 | 40946600 |
| NSUI00486 | Germany | 2137777 | 903576 | 107.7 | 2 | 2 | 28 | + | - | - | - | - | - | - | - | - | - | - | - | - | - | - | - | SAMN40946601 | 40946601 |
| NSUI00487 | Germany | 2140724 | 1244318 | 153.8 | 2 | 2 | 28 | + | - | - | - | - | - | - | - | - | - | - | - | - | - | - | - | SAMN40946602 | 40946602 |
| NSUI00488 | Germany | 2192916 | 1024651 | 119.28 | 9 | 9 | 2757 | + | - | - | - | + | - | - | - | - | - | - | - | - | - | - | - | SAMN40946603 | 40946603 |
| NSUI00489 | Germany | 2192766 | 1111318 | 138.62 | 9 | 9 | 2757 | + | - | - | - | + | - | - | - | - | - | - | - | - | - | - | - | SAMN40946604 | 40946604 |
| NSUI00490 | Germany | 2192858 | 1133511 | 139.9 | 9 | 9 | 2757 | + | - | - | - | + | - | - | - | - | - | - | - | - | - | - | - | SAMN40946605 | 40946605 |
| NSUI00491 | Germany | 2190987 | 909564 | 110.99 | 9 | 9 | 2757 | + | - | - | - | + | - | - | - | - | - | - | - | - | - | - | - | SAMN40946606 | 40946606 |
| NSUI00492 | France | 2273502 | 688282 | 83.26 | 9 | 9 | 16 | - | - | + | - | + | - | - | - | - | - | - | - | - | - | - | - | SAMN40946607 | 40946607 |
| NSUI00493 | France | 2343430 | 1252895 | 98.73 | 9 | 9 | 16 | - | - | + | - | + | - | - | - | - | - | - | - | - | - | - | - | SAMN40946608 | 40946608 |
| NSUI00494 | France | 2295295 | 747342 | 63.41 | 9 | 9 | 16 | - | - | + | - | + | - | - | - | - | - | - | - | - | - | - | - | SAMN40946609 | 40946609 |
| NSUI00495 | France | 2275957 | 1336854 | 155.19 | 9 | 9 | 16 | - | - | + | - | - | - | - | - | - | - | - | + | - | - | - | - | SAMN40946610 | 40946610 |
| NSUI00496 | France | 2272590 | 994406 | 121.98 | 9 | 9 | 16 | - | - | + | - | - | - | - | - | - | - | - | + | - | - | - | - | SAMN40946611 | 40946611 |
| NSUI00497 | France | 2104521 | 1913416 | 208.46 | 9 | 9 | 16 | - | - | - | - | - | - | - | - | - | - | - | - | - | - | - | - | SAMN40946612 | 40946612 |
| NSUI00498 | France | 1996502 | 985104 | 86.34 | 2 | 2 | 1 | - | - | - | - | - | - | - | - | - | - | - | - | - | - | - | - | SAMN40946613 | 40946613 |
| NSUI00499 | France | 2098803 | 729152 | 61.39 | 9 | 9 | 16 | - | - | - | - | - | - | - | - | - | - | - | - | - | - | - | - | SAMN40946614 | 40946614 |
| NSUI00500 | France | 1995894 | 1174871 | 97.12 | 2 | 2 | 1 | - | - | - | - | - | - | - | - | - | - | - | - | - | - | - | - | SAMN40946615 | 40946615 |
| NSUI00501 | France | 2394671 | 1131767 | 99.93 | 9 | 9 | 2773 | - | - | + | - | + | - | - | - | - | - | - | - | - | - | - | - | SAMN40946616 | 40946616 |
| NSUI00502 | France | 2348120 | 1200880 | 82.67 | 9 | 9 | 16 | - | - | + | - | + | - | - | - | - | - | - | - | - | - | - | - | SAMN40946617 | 40946617 |
| NSUI00503 | France | 2288476 | 323396 | 39.28 | 9 | 9 | 16 | - | - | + | - | - | - | - | - | - | - | - | - | - | - | - | - | SAMN40946618 | 40946618 |
| NSUI00504 | France | 2283283 | 1212824 | 97.73 | 9 | 9 | 16 | - | - | + | - | - | - | - | - | - | - | - | - | - | - | - | - | SAMN40946619 | 40946619 |
| NSUI00507 | France | 2032465 | 713301 | 63.94 | 1 | 1 | 1 | + | - | - | - | + | - | - | - | - | - | - | - | - | - | - | - | SAMN40946620 | 40946620 |
| NSUI00508 | France | 2031719 | 1245454 | 84.31 | 1 | 1 | 1 | + | - | - | - | + | - | - | - | - | - | - | - | - | - | - | - | SAMN40946621 | 40946621 |
| NSUI00509 | France | 2033824 | 1175590 | 91.58 | 1 | 1 | 1 | + | - | - | - | + | - | - | - | - | - | - | - | - | - | - | - | SAMN40946622 | 40946622 |
| NSUI00510 | France | 2030848 | 1192368 | 87.35 | 1 | 1 | 1 | + | - | - | - | + | - | - | - | - | - | - | - | - | - | - | - | SAMN40946623 | 40946623 |
| NSUI00511 | France | 1976694 | 959448 | 63.39 | 1 | 1 | 1 | - | - | - | - | - | - | - | - | - | - | - | - | - | - | - | - | SAMN40946624 | 40946624 |
| NSUI00512 | France | 1971235 | 1041517 | 72 | 1 | 1 | 1 | - | - | - | - | - | - | - | - | - | - | - | - | - | - | - | - | SAMN40946625 | 40946625 |
| NSUI00513 | Germany | 2066990 | 805678 | 74.73 | 9 | 9 | 16 | - | - | - | - | + | - | - | - | - | - | - | - | - | - | - | - | SAMN40946626 | 40946626 |
| NSUI00514 | Germany | 2058078 | 1499021 | 102.73 | 9 | 9 | 16 | - | - | - | - | + | - | - | - | - | - | - | - | - | - | - | - | SAMN40946627 | 40946627 |
| NSUI00515 | Germany | 2058567 | 1084121 | 79.8 | 9 | 9 | 16 | - | - | - | - | + | - | - | - | - | - | - | - | - | - | - | - | SAMN40946628 | 40946628 |
| NSUI00516 | Germany | 2054988 | 1415345 | 92.24 | 9 | 9 | 16 | - | - | - | - | + | - | - | - | - | - | - | - | - | - | - | - | SAMN40946629 | 40946629 |
| NSUI00517 | Germany | 2051879 | 866676 | 72.47 | 9 | 9 | 16 | - | - | - | - | + | - | - | - | - | - | - | - | - | - | - | - | SAMN40946630 | 40946630 |
| NSUI00518 | France | 2253187 | 835799 | 59.08 | 9 | 9 | 16 | - | - | + | - | + | - | - | - | - | - | - | - | - | - | - | - | SAMN40946631 | 40946631 |
| NSUI00519 | France | 2261468 | 1066928 | 85.59 | 9 | 9 | 16 | - | - | + | - | + | - | - | - | - | - | - | - | - | - | - | - | SAMN40946632 | 40946632 |
| NSUI00520 | France | 2258392 | 950949 | 77.72 | 9 | 9 | 16 | - | - | + | - | + | - | - | - | - | - | - | - | - | - | - | - | SAMN40946633 | 40946633 |
| NSUI00521 | France | 2264644 | 791572 | 64 | 9 | 9 | 16 | - | - | + | - | + | - | - | - | - | - | - | - | - | - | - | - | SAMN40946634 | 40946634 |
| NSUI00522 | France | 2151828 | 669072 | 58.64 | 2 | 2 | 28 | - | - | - | - | + | + | - | - | + | + | - | - | - | - | - | + | SAMN40946635 | 40946635 |
| NSUI00523 | France | 2153786 | 888221 | 77 | 2 | 2 | 28 | - | - | - | - | + | + | - | - | - | - | - | - | - | - | - | + | SAMN40946636 | 40946636 |
| NSUI00524 | France | 2159631 | 1004029 | 80.57 | 2 | 2 | 28 | - | - | - | - | + | + | - | - | + | + | - | - | - | - | - | + | SAMN40946637 | 40946637 |
| NSUI00525 | France | 2273251 | 1222248 | 98.28 | 9 | 9 | 1520 | - | - | + | - | - | - | - | - | - | - | - | - | - | - | - | - | SAMN40946638 | 40946638 |
| NSUI00526 | France | 2283621 | 2453610 | 153.87 | 9 | 9 | 1520 | - | - | + | - | - | - | - | - | - | - | - | - | - | - | - | - | SAMN40946639 | 40946639 |
| NSUI00527 | France | 2054080 | 600826 | 39.82 | 7 | 7 | 29 | + | - | - | - | + | - | - | - | - | - | - | - | - | - | - | - | SAMN40946640 | 40946640 |
| NSUI00528 | France | 2053861 | 611743 | 40.93 | 7 | 7 | 29 | + | - | - | - | + | - | - | - | - | - | - | - | - | - | - | - | SAMN40946641 | 40946641 |
| NSUI00529 | France | 2103921 | 512721 | 33.9 | 23 | 23 | 108 | + | - | - | - | + | - | - | - | - | - | - | - | - | - | - | - | SAMN40946642 | 40946642 |
| NSUI00530 | France | 2394330 | 498209 | 33 | 9 | 9 | 16 | - | - | + | - | + | - | - | - | - | - | - | - | - | - | - | - | SAMN40946643 | 40946643 |
| NSUI00531 | France | 2180809 | 606641 | 40.16 | 16 | 16 | 1222 | + | - | - | - | + | - | - | + | + | - | + | - | - | - | - | - | SAMN40946644 | 40946644 |
| NSUI00532 | France | 2291857 | 662208 | 44.16 | 9 | 9 | 16 | - | - | + | - | + | - | - | - | - | - | - | - | - | - | - | - | SAMN40946645 | 40946645 |
| NSUI00533 | France | 2292691 | 673420 | 45.03 | 9 | 9 | 16 | - | - | + | - | + | - | - | - | - | - | - | - | - | - | - | - | SAMN40946646 | 40946646 |
| NSUI00534 | France | 2033600 | 528688 | 36.22 | 1 | 1 | 1 | + | - | - | - | + | - | - | - | - | - | - | - | - | - | - | - | SAMN40946647 | 40946647 |
| NSUI00535 | France | 2032692 | 492201 | 33.31 | 1 | 1 | 1 | + | - | - | - | + | - | - | - | - | - | - | - | - | - | - | - | SAMN40946648 | 40946648 |
| NSUI00536 | France | 2032265 | 402978 | 27.17 | 1 | 1 | 1 | + | - | - | - | + | - | - | - | - | - | - | - | - | - | - | - | SAMN40946649 | 40946649 |
| NSUI00537 | France | 1984833 | 375204 | 25.2 | 2 | 2 | 1 | - | - | - | - | - | - | - | - | - | - | - | - | - | - | - | - | SAMN40946650 | 40946650 |
| NSUI00538 | France | 1987223 | 412409 | 27.69 | 2 | 2 | 1 | - | - | - | - | - | - | - | - | - | - | - | - | - | - | - | - | SAMN40946651 | 40946651 |
| NSUI00539 | France | 2030540 | 922653 | 72.18 | 2 | 2 | 1 | - | - | - | - | + | - | - | - | - | - | - | - | - | - | - | - | SAMN40946652 | 40946652 |
| NSUI00540 | France | 2037586 | 695297 | 61.41 | 2 | 2 | 1 | - | - | - | - | + | - | - | - | - | - | - | - | - | - | - | - | SAMN40946653 | 40946653 |
| NSUI00541 | France | 2235598 | 707658 | 61.23 | 9 | 9 | 16 | - | - | + | - | + | - | - | - | - | - | - | - | - | - | - | - | SAMN40946654 | 40946654 |
| NSUI00542 | France | 2234500 | 792292 | 66.07 | 9 | 9 | 16 | - | - | + | - | + | - | - | - | - | - | - | - | - | - | - | - | SAMN40946655 | 40946655 |
| NSUI00543 | France | 2281307 | 274147 | 27.05 | 9 | 9 | 16 | - | - | + | - | + | - | - | - | - | - | - | - | - | - | - | - | SAMN40946656 | 40946656 |
| NSUI00544 | France | 2340570 | 856205 | 71.57 | 9 | 9 | 16 | - | - | + | - | + | - | - | - | - | - | - | - | - | - | - | - | SAMN40946657 | 40946657 |
| NSUI00545 | Netherlands | 2299677 | 681428 | 54.82 | 9 | 9 | 16 | + | - | - | - | + | - | - | - | - | - | - | - | - | + | - | - | SAMN40946658 | 40946658 |
| NSUI00546 | Netherlands | 2227592 | 759801 | 61.89 | 9 | 9 | 2760 | + | - | - | - | + | - | - | - | - | - | - | - | - | - | - | - | SAMN40946659 | 40946659 |
| NSUI00547 | Netherlands | 2227216 | 955668 | 61.77 | 9 | 9 | 2760 | + | - | - | - | + | - | - | - | - | - | - | - | - | - | - | - | SAMN40946660 | 40946660 |
| NSUI00548 | Netherlands | 2304749 | 945058 | 67.73 | 9 | 9 | 16 | + | - | - | - | + | - | - | - | - | - | - | - | - | + | - | - | SAMN40946661 | 40946661 |
| NSUI00549 | Netherlands | 2325062 | 1269390 | 98.26 | 9 | 9 | 16 | + | - | - | - | + | - | - | - | - | - | - | - | - | - | - | - | SAMN40946662 | 40946662 |
| NSUI00550 | Netherlands | 2308346 | 970095 | 76.39 | 9 | 9 | 16 | + | - | - | - | + | - | - | - | - | - | - | - | - | - | - | - | SAMN40946663 | 40946663 |
| NSUI00551 | Netherlands | 2335824 | 1018916 | 81.71 | 9 | 9 | 16 | + | - | - | - | + | - | - | - | - | - | - | - | - | + | - | - | SAMN40946664 | 40946664 |
| NSUI00552 | France | 2139617 | 634268 | 54.55 | 1/2 | 1/2 | 28 | + | - | - | - | - | - | - | - | - | - | - | - | - | - | - | - | SAMN40946665 | 40946665 |
| NSUI00553 | Germany | 2029304 | 752209 | 66.26 | 1/2 | 1/2 | 1 | + | - | - | - | + | - | - | - | + | - | - | - | - | - | - | - | SAMN40946666 | 40946666 |
| NSUI00554 | Germany | 2024872 | 939143 | 77.16 | 1/2 | 1/2 | 1 | + | - | - | - | + | - | - | - | + | - | - | - | - | - | - | - | SAMN40946667 | 40946667 |
| NSUI00555 | Germany | 2136568 | 826232 | 70.42 | 7 | 7 | 29 | + | - | - | - | + | - | - | - | - | - | - | - | - | - | - | - | SAMN40946668 | 40946668 |
| NSUI00556 | Germany | 2106375 | 516243 | 34.6 | 7 | 7 | 29 | + | - | - | - | + | - | - | - | - | - | - | - | - | - | - | - | SAMN40946669 | 40946669 |
| NSUI00557 | France | 2382164 | 769459 | 66.48 | 9 | 9 | 16 | - | - | + | - | + | - | - | - | - | - | - | - | - | - | - | - | SAMN40946670 | 40946670 |
| NSUI00558 | France | 2286903 | 685888 | 59.39 | 9 | 9 | 16 | - | - | + | - | + | - | - | - | - | - | - | - | - | - | - | - | SAMN40946671 | 40946671 |
| NSUI00559 | Germany | 2158711 | 680841 | 59.51 | 7 | 7 | 29 | - | - | - | - | + | - | - | - | - | - | - | + | - | - | - | - | SAMN40946672 | 40946672 |
| NSUI00560 | Germany | 2113831 | 781200 | 66.22 | 7 | 7 | 29 | + | - | - | - | + | - | - | - | - | - | - | - | - | - | - | - | SAMN40946673 | 40946673 |
| NSUI00561 | Germany | 2028699 | 619388 | 54.68 | 1/2 | 1/2 | 1 | + | - | - | - | + | - | - | - | + | - | - | - | - | - | - | - | SAMN40946674 | 40946674 |
| NSUI00562 | Netherlands | 2034636 | 738037 | 61.53 | 2 | 2 | 1 | + | - | - | - | + | - | - | - | - | - | - | - | - | - | - | - | SAMN40946675 | 40946675 |
| NSUI00563 | Netherlands | 2034894 | 654179 | 61.53 | 2 | 2 | 1 | + | - | - | - | + | - | - | - | - | - | - | - | - | - | - | - | SAMN40946676 | 40946676 |
| NSUI00564 | Netherlands | 2037283 | 644483 | 57.31 | 2 | 2 | 1 | + | - | - | - | + | - | - | - | - | - | - | - | - | - | - | - | SAMN40946677 | 40946677 |
| NSUI00565 | Netherlands | 2037786 | 743635 | 68.9 | 2 | 2 | 1 | + | - | - | - | + | - | - | - | - | - | - | - | - | - | - | - | SAMN40946678 | 40946678 |
| NSUI00566 | Netherlands | 2037738 | 705088 | 64.5 | 2 | 2 | 1 | + | - | - | - | + | - | - | - | - | - | - | - | - | - | - | - | SAMN40946679 | 40946679 |
| NSUI00567 | Netherlands | 2037155 | 691885 | 62.46 | 2 | 2 | 1 | + | - | - | - | + | - | - | - | - | - | - | - | - | - | - | - | SAMN40946680 | 40946680 |
| NSUI00568 | France | 2193402 | 1060049 | 98.79 | 9 | 9 | 1508 | + | - | - | - | + | - | - | - | - | - | - | - | - | - | - | - | SAMN40946681 | 40946681 |
| NSUI00569 | France | 2195991 | 1002346 | 94.27 | 9 | 9 | 1508 | + | - | - | - | + | - | - | - | - | - | - | - | - | - | - | - | SAMN40946682 | 40946682 |
| NSUI00570 | France | 2153178 | 1002464 | 93.78 | 9 | 9 | 1508 | + | - | - | - | + | - | - | - | - | - | - | - | - | - | - | - | SAMN40946683 | 40946683 |
| NSUI00571 | France | 2155578 | 1092387 | 103.65 | 9 | 9 | 1508 | + | - | - | - | + | - | - | - | - | - | - | - | - | - | - | - | SAMN40946684 | 40946684 |
| NSUI00572 | France | 2245256 | 1342679 | 126.23 | 9 | 9 | 16 | + | - | - | - | + | - | - | - | - | - | - | - | - | - | - | - | SAMN40946685 | 40946685 |
| NSUI00573 | France | 2293306 | 1997742 | 169.6 | 9 | 9 | 16 | + | - | - | - | + | - | - | - | - | - | - | - | - | - | - | - | SAMN40946686 | 40946686 |
| NSUI00574 | France | 2038218 | 1254077 | 101.05 | 9 | 9 | 1521 | - | - | - | - | - | - | - | - | - | - | - | - | - | - | - | - | SAMN40946687 | 40946687 |
| NSUI00575 | France | 2031793 | 917690 | 83.81 | 9 | 9 | 1521 | - | - | - | - | - | - | - | - | - | - | - | - | - | - | - | - | SAMN40946688 | 40946688 |
| NSUI00576 | France | 2269799 | 2540076 | 191.06 | 9 | 9 | 16 | + | - | - | - | + | - | - | - | - | - | - | - | - | - | - | - | SAMN40946689 | 40946689 |
| NSUI00577 | France | 2262087 | 1175319 | 102.64 | 9 | 9 | 16 | + | - | - | - | + | - | - | - | - | - | - | - | - | - | - | - | SAMN40946690 | 40946690 |
| NSUI00578 | France | 2038255 | 1318666 | 111.08 | 1 | 1 | 1 | + | - | - | - | + | - | - | - | - | - | - | - | - | - | - | - | SAMN40946691 | 40946691 |
| NSUI00579 | France | 2036866 | 1530076 | 146.41 | 1 | 1 | 1 | + | - | - | - | + | - | - | - | - | - | - | - | - | - | - | - | SAMN40946692 | 40946692 |
| NSUI00580 | Germany | 2190709 | 2477863 | 187.12 | 3 | 3 | 2791 | - | - | - | - | - | - | - | - | - | - | - | - | - | - | - | - | SAMN40946693 | 40946693 |
| NSUI00581 | Germany | 2025672 | 1026584 | 89.89 | 4 | 4 | 17 | - | - | - | - | - | - | - | - | - | - | - | - | + | - | - | - | SAMN40946694 | 40946694 |
| NSUI00582 | France | 2205437 | 497122 | 30.25 | 18 | 18 | 2793 | - | - | - | - | - | - | - | - | - | - | - | - | - | - | - | - | SAMN40946695 | 40946695 |
| NSUI00583 | France | 2330904 | 2328320 | 189.43 | 18 | 18 | 2793 | - | - | - | - | - | - | - | - | - | - | - | - | - | - | - | - | SAMN40946696 | 40946696 |
| NSUI00584 | France | 2130129 | 2574669 | 189.65 | 2 | 2 | 28 | - | - | + | - | + | - | - | - | - | - | - | - | - | - | - | - | SAMN40946697 | 40946697 |
| NSUI00585 | France | 2129548 | 2669496 | 191.61 | 2 | 2 | 28 | - | - | + | - | + | - | - | - | - | - | - | - | - | - | - | - | SAMN40946698 | 40946698 |
| NSUI00587 | Belgium | 2097890 | 861742 | 53.99 | 7 | 7 | 29 | + | - | - | - | + | + | - | + | + | - | - | - | - | - | - | + | SAMN40946699 | 40946699 |
| NSUI00588 | Belgium | 2345215 | 1965699 | 162.33 | 9 | 9 | 16 | + | - | - | - | + | - | - | - | - | - | - | - | - | - | - | - | SAMN40946700 | 40946700 |
| NSUI00589 | Belgium | 2345969 | 2635673 | 205.69 | 9 | 9 | 16 | + | - | - | - | + | - | - | - | - | - | - | - | - | - | - | - | SAMN40946701 | 40946701 |
| NSUI00590 | Belgium | 2308284 | 1875501 | 172.99 | 9 | 9 | 16 | + | - | - | - | + | - | - | - | - | - | - | - | - | - | - | - | SAMN40946702 | 40946702 |
| NSUI00591 | Belgium | 2309597 | 2270689 | 205.97 | 9 | 9 | 16 | + | - | - | - | + | - | - | - | - | - | - | - | - | - | - | - | SAMN40946703 | 40946703 |
| NSUI00592 | France | 2041971 | 1937870 | 168.77 | 2 | 2 | 1 | + | - | - | - | + | - | - | - | - | - | - | - | - | - | - | - | SAMN40946704 | 40946704 |
| NSUI00593 | France | 2035615 | 1023958 | 86.57 | 2 | 2 | 1 | + | - | - | - | + | - | - | - | - | - | - | - | - | - | - | - | SAMN40946705 | 40946705 |
| NSUI00594 | France | 2392848 | 2189058 | 183.81 | 9 | 9 | 16 | - | - | + | - | + | - | - | - | - | - | - | - | - | - | - | - | SAMN40946706 | 40946706 |
| NSUI00595 | France | 2394833 | 2322625 | 200.43 | 9 | 9 | 16 | - | - | + | - | + | - | - | - | - | - | - | - | - | - | - | - | SAMN40946707 | 40946707 |
| NSUI00596 | France | 1981721 | 169281 | 12.18 | 9 | 9 | 16 | - | - | + | - | - | - | - | - | - | - | - | - | - | - | - | - | SAMN40946708 | 40946708 |
| NSUI00597 | Netherlands | 2037173 | 879962 | 54.85 | 2 | 2 | 1 | + | - | - | - | + | - | - | - | - | - | - | - | - | - | - | - | SAMN40946709 | 40946709 |
| NSUI00598 | Netherlands | 2033165 | 851607 | 52.44 | 2 | 2 | 1 | + | - | - | - | - | - | - | - | - | - | - | - | - | - | - | - | SAMN40946710 | 40946710 |
| NSUI00599 | Netherlands | 2148285 | 741032 | 45.37 | 9 | 9 | 16 | - | - | + | - | - | - | - | - | - | - | - | - | - | - | - | - | SAMN40946711 | 40946711 |
| NSUI00600 | Netherlands | 2126507 | 348262 | 21.15 | 9 | 9 | 16 | - | - | + | - | - | - | - | - | - | - | - | - | - | - | - | - | SAMN40946712 | 40946712 |
| NSUI00601 | France | 2009404 | 607810 | 37.78 | 2 | 2 | 1 | + | - | - | - | - | - | - | - | - | - | - | - | - | - | - | - | SAMN40946713 | 40946713 |
| NSUI00602 | France | 1997472 | 631045 | 39.18 | 2 | 2 | 1 | - | - | - | - | + | - | - | - | - | - | - | - | - | - | - | - | SAMN40946714 | 40946714 |
| NSUI00603 | France | 2013475 | 724600 | 45.92 | 2 | 2 | 1 | - | - | - | - | + | - | - | - | - | - | - | - | - | - | - | - | SAMN40946715 | 40946715 |
| NSUI00604 | France | 2022304 | 840181 | 52.14 | 2 | 2 | 1 | - | - | - | - | + | - | - | - | - | - | - | - | - | - | - | - | SAMN40946716 | 40946716 |
| NSUI00605 | France | 2030926 | 835829 | 50.36 | 2 | 2 | 1 | - | + | - | - | - | - | - | - | - | - | - | - | - | - | - | - | SAMN40946717 | 40946717 |
| NSUI00606 | France | 1963851 | 866906 | 49.32 | 2 | 2 | 1 | - | - | - | - | - | - | - | - | - | - | - | - | - | - | - | - | SAMN40946718 | 40946718 |
| NSUI00607 | France | 2112305 | 1106759 | 59.78 | UT | UT | 2796 | + | - | - | - | - | - | - | - | - | - | - | - | - | - | - | - | SAMN40946719 | 40946719 |
| NSUI00608 | France | 2052591 | 656941 | 41.24 | 9 | 9 | 16 | - | - | - | - | - | - | - | - | - | - | - | - | - | - | - | - | SAMN40946720 | 40946720 |
| NSUI00609 | France | 2049739 | 1153468 | 70.57 | 9 | 9 | 16 | - | - | - | - | - | - | - | - | - | - | - | - | - | - | - | - | SAMN40946721 | 40946721 |
| NSUI00610 | France | 2182914 | 615889 | 38.08 | 9 | 9 | 1520 | - | - | + | - | - | - | - | - | - | - | - | + | - | - | - | - | SAMN40946722 | 40946722 |
| NSUI00611 | France | 2254468 | 651128 | 42.26 | 9 | 9 | 1520 | - | - | + | - | - | - | - | - | - | - | - | + | - | - | - | - | SAMN40946723 | 40946723 |
| NSUI00612 | France | 2029298 | 574514 | 37.1 | 2 | 2 | 1 | + | - | - | - | + | - | - | + | + | - | + | - | - | - | - | - | SAMN40946724 | 40946724 |
| NSUI00613 | France | 2032420 | 440583 | 30.34 | 2 | 2 | 1 | + | - | - | - | + | - | - | + | + | - | + | - | - | - | - | - | SAMN40946725 | 40946725 |
| NSUI00614 | France | 2129902 | 877756 | 56.17 | 9 | 9 | 16 | - | - | - | - | - | - | - | - | - | - | - | - | - | - | - | - | SAMN40946726 | 40946726 |
| NSUI00615 | Germany | 2044170 | 1005497 | 64.38 | 4 | 4 | 17 | - | - | - | - | - | - | - | - | - | - | - | - | - | - | - | - | SAMN40946727 | 40946727 |
| NSUI00616 | Germany | 2041813 | 593076 | 37.21 | 4 | 4 | 17 | - | - | - | - | - | - | - | - | - | - | - | - | - | - | - | - | SAMN40946728 | 40946728 |
| NSUI00617 | Germany | 2020283 | 552510 | 35.86 | 4 | 4 | 17 | - | - | - | - | - | - | - | - | - | - | - | - | - | - | - | - | SAMN40946729 | 40946729 |
| NSUI00618 | Germany | 2047954 | 666404 | 42.87 | 4 | 4 | 17 | - | - | - | - | - | - | - | - | - | - | - | - | - | - | - | - | SAMN40946730 | 40946730 |
| NSUI00619 | Netherlands | 2000747 | 582977 | 38.15 | 2 | 2 | 1 | + | - | - | - | + | - | - | - | - | - | - | - | - | - | - | - | SAMN40946731 | 40946731 |
| NSUI00620 | Netherlands | 2005688 | 645157 | 41.85 | 2 | 2 | 1 | + | - | - | - | + | - | - | - | - | - | - | - | - | - | - | - | SAMN40946732 | 40946732 |
| NSUI00621 | Netherlands | 2020122 | 1061011 | 66.45 | 2 | 2 | 1 | + | - | - | - | + | - | - | - | - | - | - | - | - | - | - | - | SAMN40946733 | 40946733 |
| NSUI00622 | Netherlands | 2026497 | 778232 | 49.31 | 2 | 2 | 1 | + | - | - | - | + | - | - | - | - | - | - | - | - | - | - | - | SAMN40946734 | 40946734 |
| NSUI00623 | Netherlands | 2043620 | 623264 | 39.51 | 2 | 2 | 1 | + | - | - | - | + | - | - | - | - | - | - | - | - | - | - | - | SAMN40946735 | 40946735 |
| NSUI00624 | Netherlands | 2041406 | 915335 | 55.34 | 2 | 2 | 1 | + | - | - | - | + | - | - | - | - | - | - | - | - | - | - | - | SAMN40946736 | 40946736 |
| NSUI00625 | Netherlands | 2040330 | 978063 | 58.03 | 2 | 2 | 1 | + | - | - | - | + | - | - | - | - | - | - | - | - | - | - | - | SAMN40946737 | 40946737 |
| NSUI00626 | Netherlands | 2043752 | 610692 | 37.18 | 2 | 2 | 1 | + | - | - | - | + | - | - | - | - | - | - | - | - | - | - | - | SAMN40946738 | 40946738 |
| NSUI00627 | France | 2006830 | 993218 | 58.93 | 2 | 2 | 2753 | + | - | - | - | - | - | - | - | - | - | - | - | - | - | - | - | SAMN40946739 | 40946739 |
| NSUI00628 | France | 2138660 | 929610 | 54.51 | 9 | 9 | 16 | - | - | + | - | - | - | - | - | - | - | - | - | - | - | - | - | SAMN40946740 | 40946740 |
| NSUI00629 | France | 2133077 | 898967 | 52.98 | 9 | 9 | 16 | - | - | + | - | - | - | - | - | - | - | - | - | - | - | - | - | SAMN40946741 | 40946741 |
| NSUI00630 | France | 2040461 | 677831 | 41.09 | 9 | 9 | 16 | - | - | - | - | - | - | - | - | - | - | - | - | - | - | - | - | SAMN40946742 | 40946742 |
| NSUI00632 | France | 2011053 | 607773 | 38.75 | 2 | 2 | 2753 | + | - | - | - | - | - | - | - | - | - | - | - | - | - | - | - | SAMN40946743 | 40946743 |
| NSUI00633 | France | 2423605 | 706969 | 43.19 | UT | UT | 2798 | + | - | - | + | + | - | - | + | - | - | + | - | - | - | - | - | SAMN40946744 | 40946744 |
| NSUI00634 | France | 2040929 | 609600 | 37.53 | 7 | 7 | 29 | + | - | - | - | + | - | - | - | - | - | - | - | - | - | - | - | SAMN40946745 | 40946745 |
| NSUI00635 | France | 2028717 | 830463 | 49.73 | 7 | 7 | 29 | + | - | - | - | + | - | - | - | - | - | - | - | - | - | - | - | SAMN40946746 | 40946746 |
| NSUI00636 | France | 2024050 | 1014331 | 63.07 | 2 | 2 | 1 | + | - | - | - | + | - | - | + | + | - | + | - | - | - | - | - | SAMN40946747 | 40946747 |
| NSUI00637 | France | 2083805 | 573879 | 34.98 | 9 | 9 | 1508 | + | - | - | - | + | - | - | - | - | - | - | - | - | - | - | - | SAMN40946748 | 40946748 |
| NSUI00638 | France | 2112843 | 559969 | 35.86 | 9 | 9 | 1508 | + | - | - | - | + | - | - | - | - | - | - | - | - | - | - | - | SAMN40946749 | 40946749 |
| NSUI00639 | France | 2332290 | 555583 | 34.77 | 9 | 9 | 16 | - | - | + | - | + | - | - | - | - | - | - | - | - | - | - | - | SAMN40946750 | 40946750 |
| NSUI00640 | France | 2351226 | 668311 | 42.91 | 9 | 9 | 16 | - | - | + | - | + | - | - | - | - | - | - | - | - | - | - | - | SAMN40946751 | 40946751 |
| NSUI00641 | France | 2329022 | 432740 | 27.96 | 9 | 9 | 16 | - | - | + | - | + | - | - | - | - | - | - | - | - | - | - | - | SAMN40946752 | 40946752 |
| NSUI00643 | Hungary | 2100614 | 727227 | 44.89 | UT | UT | 28 | - | - | - | - | + | + | - | - | + | - | - | - | - | - | - | + | SAMN40946753 | 40946753 |
| NSUI00644 | Hungary | 2098763 | 665078 | 40.63 | UT | UT | 28 | - | - | - | - | + | + | - | - | + | - | - | - | - | - | - | + | SAMN40946754 | 40946754 |
| NSUI00645 | Netherlands | 2246171 | 877529 | 60.37 | 2 | 2 | 20 | - | + | - | - | - | - | - | - | - | - | - | + | - | - | - | - | SAMN40946755 | 40946755 |
| NSUI00646 | Netherlands | 2246073 | 491010 | 33.62 | 2 | 2 | 20 | - | + | - | - | - | - | - | - | - | - | - | + | - | - | - | - | SAMN40946756 | 40946756 |
| NSUI00647 | Netherlands | 2207589 | 732204 | 49.7 | 9 | 9 | 16 | - | - | + | - | - | - | - | - | - | - | - | - | - | - | - | - | SAMN40946757 | 40946757 |
| NSUI00648 | Netherlands | 2208675 | 769879 | 52.78 | 9 | 9 | 16 | - | - | + | - | - | - | - | - | - | - | - | - | - | - | - | - | SAMN40946758 | 40946758 |
| NSUI00649 | Netherlands | 2207356 | 388152 | 26.52 | 9 | 9 | 16 | - | - | + | - | - | - | - | - | - | - | - | - | - | - | - | - | SAMN40946759 | 40946759 |
| NSUI00650 | Netherlands | 2191878 | 565775 | 38.7 | 9 | 9 | 16 | - | - | + | - | - | - | - | - | - | - | - | - | - | - | - | - | SAMN40946760 | 40946760 |
| NSUI00651 | Netherlands | 2207256 | 358765 | 24.37 | 9 | 9 | 16 | - | - | + | - | - | - | - | - | - | - | - | - | - | - | - | - | SAMN40946761 | 40946761 |
| NSUI00652 | Netherlands | 2208418 | 456186 | 31.24 | 9 | 9 | 16 | - | - | + | - | - | - | - | - | - | - | - | - | - | - | - | - | SAMN40946762 | 40946762 |
| NSUI00653 | France | 2125679 | 561499 | 38.38 | 9 | 9 | 1521 | - | - | + | - | - | + | - | - | - | - | - | - | - | - | - | + | SAMN40946763 | 40946763 |
| NSUI00654 | France | 2126265 | 586653 | 40.26 | 9 | 9 | 1521 | - | - | + | - | - | + | - | - | - | - | - | - | - | - | - | + | SAMN40946764 | 40946764 |
| NSUI00655 | Netherlands | 2033461 | 766037 | 52.47 | 2 | 2 | 1 | + | - | - | - | + | - | - | - | - | - | - | - | - | - | - | - | SAMN40946765 | 40946765 |
| NSUI00656 | Netherlands | 2032630 | 387278 | 26.43 | 2 | 2 | 1 | + | - | - | - | + | - | - | - | - | - | - | - | - | - | - | - | SAMN40946766 | 40946766 |
| NSUI00657 | Netherlands | 2033435 | 939714 | 64.27 | 2 | 2 | 1 | + | - | - | - | + | - | - | - | - | - | - | - | - | - | - | - | SAMN40946767 | 40946767 |
| NSUI00658 | Netherlands | 2031971 | 600941 | 41.08 | 2 | 2 | 1 | + | - | - | - | + | - | - | - | - | - | - | - | - | - | - | - | SAMN40946768 | 40946768 |
| NSUI00660 | Netherlands | 2108301 | 705389 | 48.02 | 7 | 7 | 29 | + | - | - | - | + | - | - | - | - | - | - | - | - | - | - | - | SAMN40946769 | 40946769 |
| NSUI00661 | Netherlands | 2107193 | 634353 | 43.52 | 7 | 7 | 29 | + | - | - | - | + | - | - | - | - | - | - | - | - | - | - | - | SAMN40946770 | 40946770 |
| NSUI00662 | Netherlands | 2108060 | 459920 | 31.27 | 7 | 7 | 29 | + | - | - | - | + | - | - | - | - | - | - | - | - | - | - | - | SAMN40946771 | 40946771 |
| NSUI00664 | Netherlands | 2293998 | 501620 | 34.3 | 9 | 9 | 16 | - | - | - | - | - | - | - | - | - | - | - | - | - | - | + | - | SAMN40946772 | 40946772 |
| NSUI00667 | UK | 2217904 | 611854 | 41.68 | 8 | 8 | 87 | - | - | + | - | + | - | - | - | - | - | - | + | - | - | - | - | SAMN40946773 | 40946773 |
| NSUI00668 | UK | 1970002 | 482926 | 33.05 | 1 | 1 | 1 | - | - | - | - | - | - | - | - | - | - | - | - | - | - | - | - | SAMN40946774 | 40946774 |
| NSUI00671 | UK | 2100349 | 461292 | 30.6 | 9 | 9 | 2774 | - | + | - | - | + | - | - | - | - | - | - | - | - | - | - | - | SAMN40946775 | 40946775 |
| NSUI00672 | UK | 2045940 | 623897 | 42.54 | 2 | 2 | 2768 | + | - | - | - | + | - | - | - | - | - | - | - | - | - | - | - | SAMN40946776 | 40946776 |
| NSUI00673 | UK | 2045803 | 570447 | 38.95 | 2 | 2 | 2768 | + | - | - | - | + | - | - | - | - | - | - | - | - | - | - | - | SAMN40946777 | 40946777 |
| NSUI00674 | Netherlands | 2146929 | 552737 | 37.67 | 10 | 10 | 2769 | + | - | - | - | + | - | - | + | + | - | + | - | - | - | - | - | SAMN40946778 | 40946778 |
| NSUI00675 | Netherlands | 2148113 | 829427 | 56.92 | 10 | 10 | 2769 | + | - | - | - | + | - | - | + | + | - | + | - | - | - | - | - | SAMN40946779 | 40946779 |
| NSUI00676 | Netherlands | 2148574 | 492582 | 33.69 | 10 | 10 | 2769 | + | - | - | - | + | - | - | + | + | - | + | - | - | - | - | - | SAMN40946780 | 40946780 |
| NSUI00677 | Netherlands | 2045241 | 458131 | 31.22 | 2 | 2 | 1 | + | - | - | - | + | - | - | - | - | - | - | - | - | - | - | - | SAMN40946781 | 40946781 |
| NSUI00678 | Netherlands | 2044682 | 702753 | 45.57 | 2 | 2 | 1 | + | - | - | - | + | - | - | - | - | - | - | - | - | - | - | - | SAMN40946782 | 40946782 |
| NSUI00679 | Netherlands | 2044270 | 526763 | 35.42 | 2 | 2 | 1 | + | - | - | - | + | - | - | - | - | - | - | - | - | - | - | - | SAMN40946783 | 40946783 |
| NSUI00680 | Netherlands | 2147506 | 428582 | 29.3 | 10 | 10 | 2769 | + | - | - | - | + | - | - | + | + | - | + | - | - | - | - | - | SAMN40946784 | 40946784 |
| NSUI00681 | Netherlands | 2045572 | 595088 | 40.03 | 2 | 2 | 1 | + | - | - | - | + | - | - | - | - | - | - | - | - | - | - | - | SAMN40946785 | 40946785 |
| NSUI00682 | Netherlands | 2169749 | 834626 | 56.9 | 9 | 9 | 819 | + | - | - | - | + | - | - | + | + | - | + | - | - | - | - | - | SAMN40946786 | 40946786 |
| NSUI00683 | Netherlands | 2158044 | 499466 | 34.2 | 9 | 9 | 819 | + | - | - | - | + | - | - | + | + | - | + | - | - | - | - | - | SAMN40946787 | 40946787 |
| NSUI00684 | France | 2055287 | 599294 | 40.68 | 2 | 2 | 1 | - | - | - | - | + | - | - | - | - | - | - | - | - | - | - | - | SAMN40946788 | 40946788 |
| NSUI00685 | France | 2055021 | 418873 | 27.84 | 2 | 2 | 1 | - | - | - | - | + | - | - | - | - | - | - | - | - | - | - | - | SAMN40946789 | 40946789 |
| NSUI00686 | France | 2127237 | 597805 | 40.5 | 7 | 7 | 2771 | + | - | - | - | + | - | - | - | - | - | - | - | - | - | - | - | SAMN40946790 | 40946790 |
| NSUI00688 | UK | 2101217 | 257259 | 17.6 | 9 | 9 | 2767 | - | - | + | - | - | - | - | - | - | - | - | - | - | - | - | - | SAMN40946791 | 40946791 |
| NSUI00689 | UK | 2183651 | 479901 | 32.88 | 8 | 8 | 2775 | - | - | - | - | - | - | - | - | - | - | - | - | - | - | - | - | SAMN40946792 | 40946792 |
| NSUI00691 | UK | 2071500 | 510621 | 34.88 | 2 | 2 | 1 | - | - | - | - | - | - | - | - | - | - | - | - | - | - | - | - | SAMN40946793 | 40946793 |
| NSUI00692 | UK | 2127784 | 638924 | 43.28 | 1/2 | 1/2 | 28 | + | - | - | - | + | - | - | - | - | - | - | - | - | - | - | - | SAMN40946794 | 40946794 |
| NSUI00694 | UK | 2023054 | 612108 | 41.31 | 2 | 2 | 2 | + | - | - | - | - | - | - | - | - | - | - | - | - | - | - | - | SAMN40946795 | 40946795 |
| NSUI00695 | UK | 2024232 | 473886 | 31.93 | 2 | 2 | 2 | + | - | - | - | - | - | - | - | - | - | - | - | - | - | - | - | SAMN40946796 | 40946796 |
| NSUI00697 | UK | 2035373 | 296171 | 19.56 | 2 | 2 | 2 | + | - | - | - | - | - | - | - | - | - | - | - | - | - | - | - | SAMN40946797 | 40946797 |
| NSUI00698 | UK | 2035072 | 303463 | 20.39 | 2 | 2 | 2 | + | - | - | - | - | - | - | - | - | - | - | - | - | - | - | - | SAMN40946798 | 40946798 |
| NSUI00699 | UK | 2024235 | 294164 | 19.72 | 2 | 2 | 2 | + | - | - | - | - | - | - | - | - | - | - | - | - | - | - | - | SAMN40946799 | 40946799 |
| NSUI00700 | France | 1969185 | 295836 | 19.96 | 1 | 1 | 1 | - | - | - | - | - | - | - | - | - | - | - | - | - | - | - | - | SAMN40946800 | 40946800 |
| NSUI00701 | France | 1969806 | 534952 | 35.83 | 1 | 1 | 1 | - | - | - | - | - | - | - | - | - | - | - | - | - | - | - | - | SAMN40946801 | 40946801 |
| NSUI00702 | France | 2132242 | 421712 | 28.74 | 3 | 3 | 94 | + | - | - | - | + | - | - | - | - | - | - | - | - | - | - | - | SAMN40946802 | 40946802 |
| NSUI00704 | France | 2124593 | 250809 | 17.19 | 9 | 9 | 1521 | - | - | + | - | - | + | - | - | - | - | - | - | - | - | - | + | SAMN40946803 | 40946803 |
| NSUI00705 | France | 2124995 | 627817 | 42.97 | 9 | 9 | 1521 | - | - | + | - | - | + | - | - | - | - | - | - | - | - | - | + | SAMN40946804 | 40946804 |
| NSUI00706 | Spain | 2047562 | 750682 | 51.56 | 1 | 1 | 1552 | + | - | - | - | + | - | - | - | - | - | - | - | - | - | - | - | SAMN40946805 | 40946805 |
| NSUI00707 | Spain | 2047975 | 889224 | 61.21 | 1 | 1 | 1552 | + | - | - | - | + | - | - | - | - | - | - | - | - | - | - | - | SAMN40946806 | 40946806 |
| NSUI00708 | UK | 1980651 | 234600 | 15.94 | 1 | 1 | 1 | - | - | - | - | - | - | - | - | - | - | - | - | - | - | - | - | SAMN40946807 | 40946807 |
| NSUI00709 | France | 2165628 | 635518 | 43.11 | 9 | 9 | 1521 | - | - | + | - | - | + | - | - | - | - | - | - | - | - | - | + | SAMN40946808 | 40946808 |
| NSUI00710 | France | 2085291 | 450471 | 30.36 | 2 | 2 | 1 | - | - | - | - | - | - | - | - | - | - | - | - | - | - | - | - | SAMN40946809 | 40946809 |
| NSUI00711 | France | 2084964 | 313594 | 20.85 | 2 | 2 | 1 | - | - | - | - | - | - | - | - | - | - | - | - | - | - | - | - | SAMN40946810 | 40946810 |
| NSUI00713 | France | 2047873 | 466458 | 31.16 | 2 | 2 | 1 | - | - | - | - | + | - | - | - | + | - | - | - | - | - | - | - | SAMN40946811 | 40946811 |
| NSUI00714 | France | 2048043 | 270841 | 18.04 | 2 | 2 | 1 | - | - | - | - | + | - | - | - | + | - | - | - | - | - | - | - | SAMN40946812 | 40946812 |

^a^ Sum of all *de novo* assembled contigs for each isolate.

^b^ Calculated based on an average genome size for *S. suis* of 2 200 000 bp.

^c^ ST: sequence type as determined by in silico-based multilocus sequence typing

^d^ National Center for Biotechnology Information BioSample (<https://www.ncbi.nlm.nih.gov/biosample/>) accession number.

^e^ National Center for Biotechnology Information Sequence Read Archive (<https://www.ncbi.nlm.nih.gov/sra/>) accession number.
