# Supplementary figures and images for: Molecular characterization of Streptococcus suis isolates recovered from diseased pigs in Europe
Source: Vet Res. 2024 Sep 27;55:117. doi: 10.1186/s13567-024-01366-y (PMC11429987; doi:10.1186/s13567-024-01366-y)

A

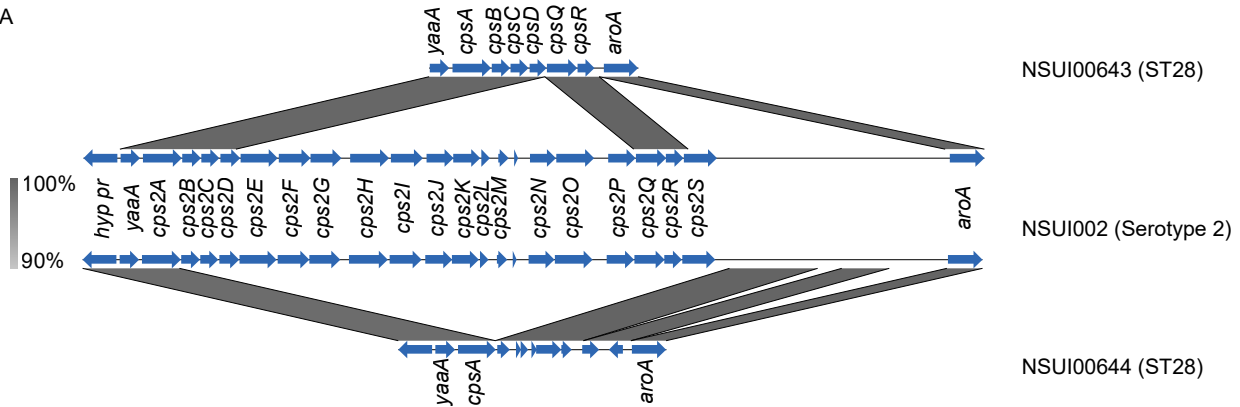

B

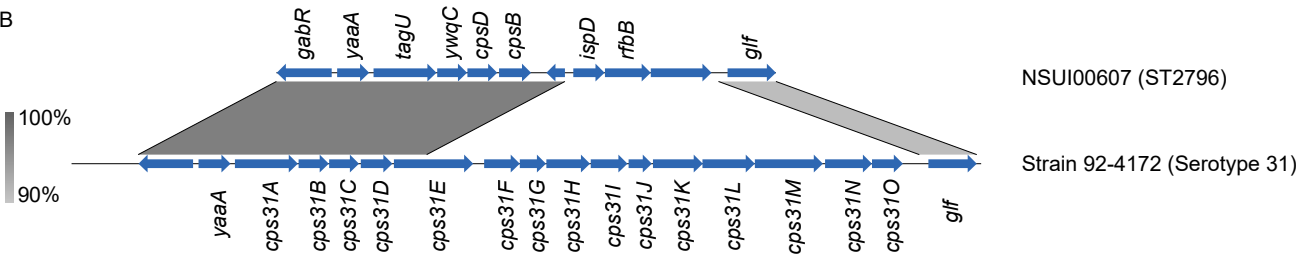

C

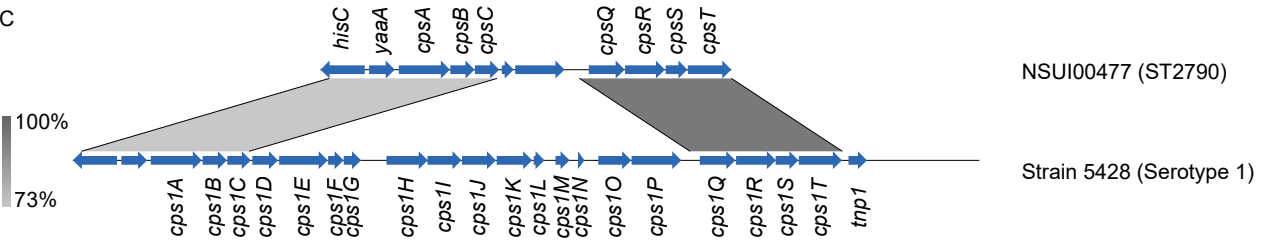

D

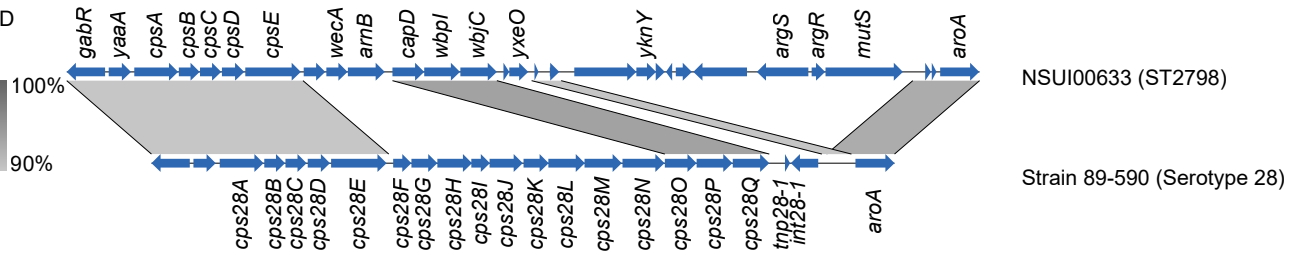

Supplement: Supplementary file 2 — Additional file 2. Comparative architecture of cps loci in untypable Streptococcus suis isolates. Illustrated is the genetic organization of the cps loci of the five S. suis untypable isolates, including a comparison to appropriate reference cps loci sequences. Percentage identity to reference sequences is indicated. A) ST28 isolates NSUI00643 and NSUI00644, both recovered in Hungary, possessed some but lacked several key cps genes found in the reference ST28 serotype 2 strain NSUI002 (GenBank accession number CP011419.1). B) ST2796 isolate NSUI00607, recovered in France, possessed some but lacked several key cps genes found in serotype 31 strain 92-4172 (GenBank accession number AB737835.1). C) ST2790 isolate NSUI00477, recovered in the Netherlands, possessed some but lacked several key cps genes found in reference serotype 1 strain 5428 (GenBank accession number JF273644.1). D) ST2798 isolate NSUI00633, recovered in France, possessed some but lacked several key cps genes found in reference serotype 28 strain 89-590 (GenBank accession number AB737832.1). [file 13567_2024_1366_MOESM2_ESM.pdf]

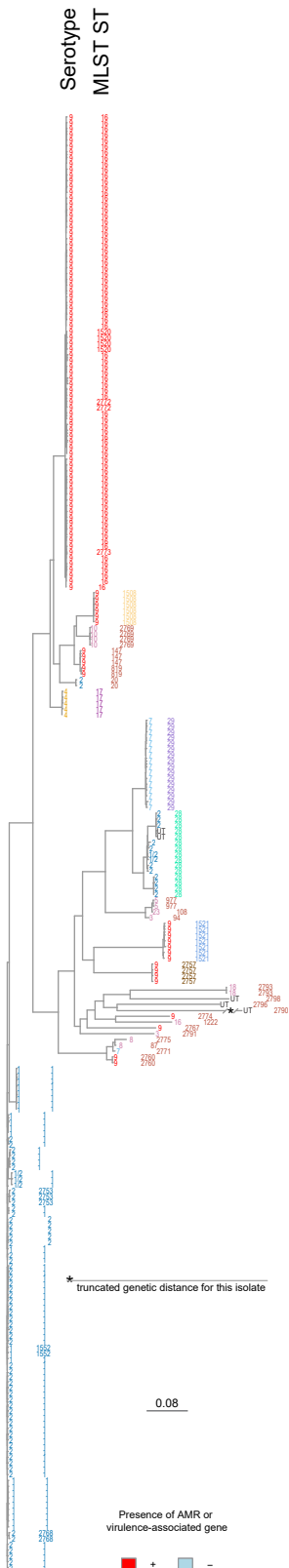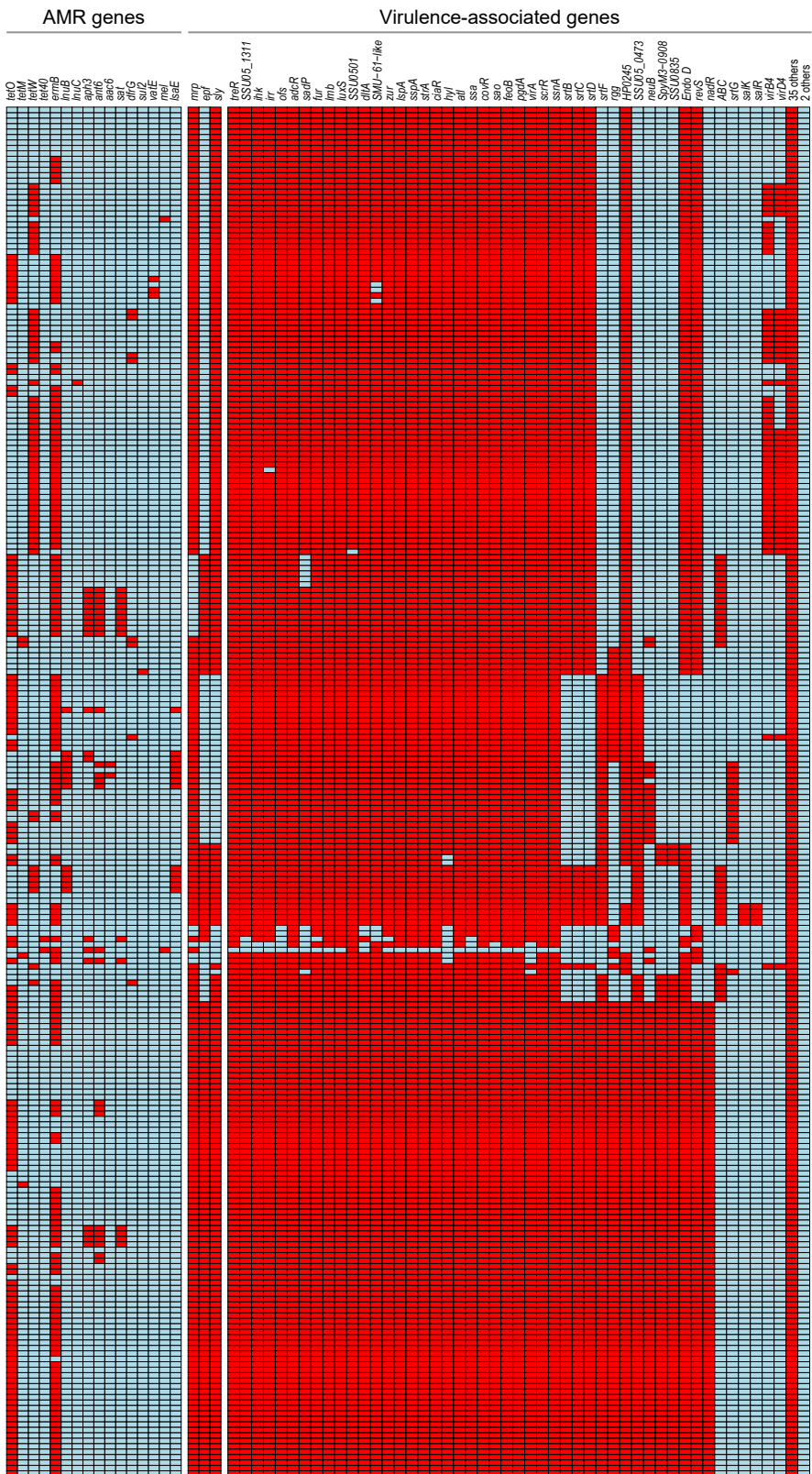

Supplement: Supplementary file 4 — Additional file 4. Phylogenetic relationships based on core-genome single-nucleotide polymorphisms (SNPs) and genomic traits of the 251 Streptococcus suis isolates using a different reference genome. This figure presents a maximum-likelihood phylogenetic tree (left panel), constructed using 8,611 non-redundant core-genome SNP loci identified relative to the genome sequence of the ST16 serotype 9 reference strain GD-0088. This analysis confirms the findings depicted in Figure 3, using a different reference to provide comparative insights. The tree highlights several distinct clades, emphasizing the genetic diversity among the isolates. For reference, the serotype of each isolate, along with the genotypes determined by multilocus sequence typing (MLST), are annotated along the tree, showing their association with specific genomic clades. The right panel depicts the presence (in purple) or absence (in light blue) of antimicrobial resistance (AMR) genes and virulence-associated genes (VAGs), as determined from the whole-genome sequences of each isolate. “UT” denotes an untypable isolate. [file 13567_2024_1366_MOESM4_ESM.pdf]

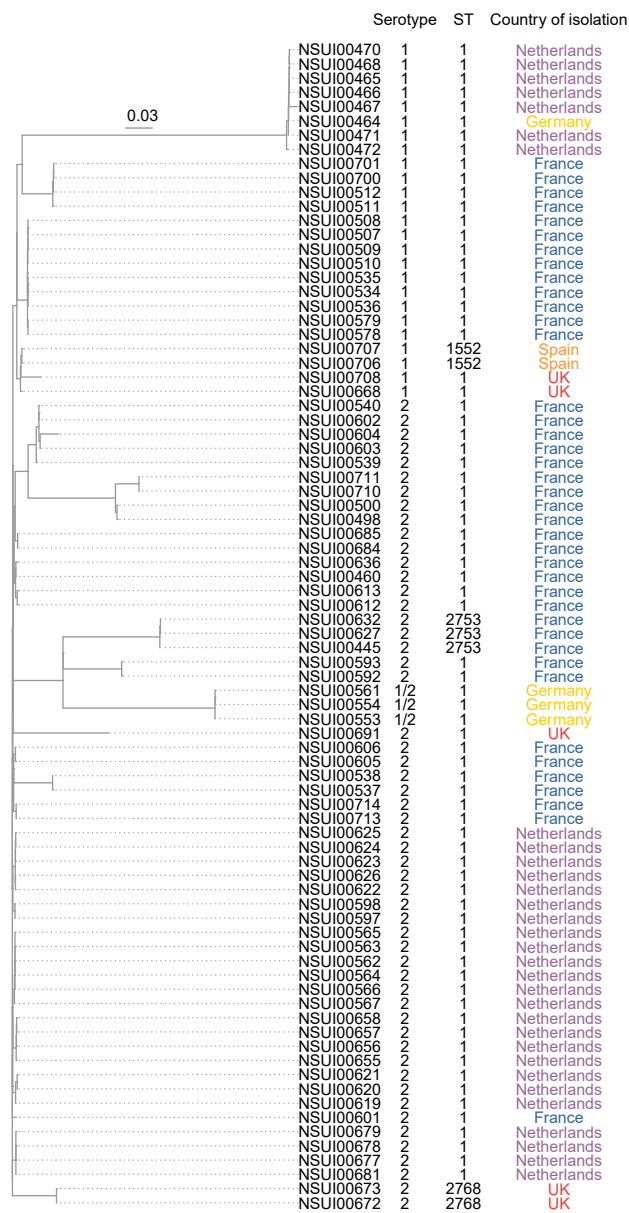

Supplement: Supplementary file 5 — Additional file 5. Inferred genetic relationships based on core-genome analysis among the 82 Streptococcus suis isolates investigated in this study belonging to sequence type (ST) 1 and closely related STs. The maximum likelihood phylogenetic tree was constructed from 6,272 non-redundant core-genome SNP loci identified relative to the genome sequence of the ST1 serotype 2 reference strain P1/7. Each isolate is uniquely identified (e.g., NSUI00470) and annotated with its serotype and ST. The tree includes isolates from France, Germany, the Netherlands, Spain, and the United Kingdom, with each country represented by a specific color. This illustrates the geographic distribution of the strains and highlights that patterns of strain diversification have a strong geographic signature. [file 13567_2024_1366_MOESM5_ESM.pdf]

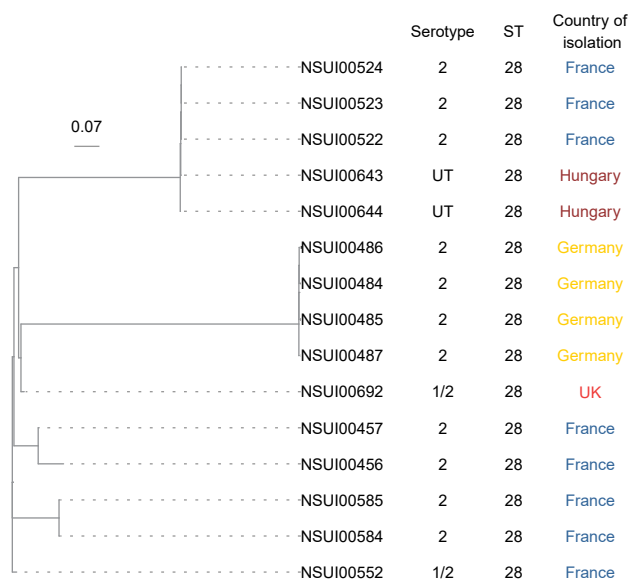

Supplement: Supplementary file 6 — Additional file 6. Inferred genetic relationships based on core-genome analysis among the 15 Streptococcus suis isolates belonging to sequence type 28 investigated in this study. The maximum likelihood phylogenetic tree was constructed from 8906 non-redundant core-genome SNP loci identified relative to the genome sequence of the ST28 serotype 2 reference strain NSUI002 (GenBank Accession number CP011419.1). Each isolate is uniquely identified (e.g., NSUI00524) and annotated with its 24 serotype and ST. The tree includes isolates from France, Germany, Hungary, and the United Kingdom, with each country represented by a specific color. “UT” denotes an untypable. [file 13567_2024_1366_MOESM6_ESM.pdf]

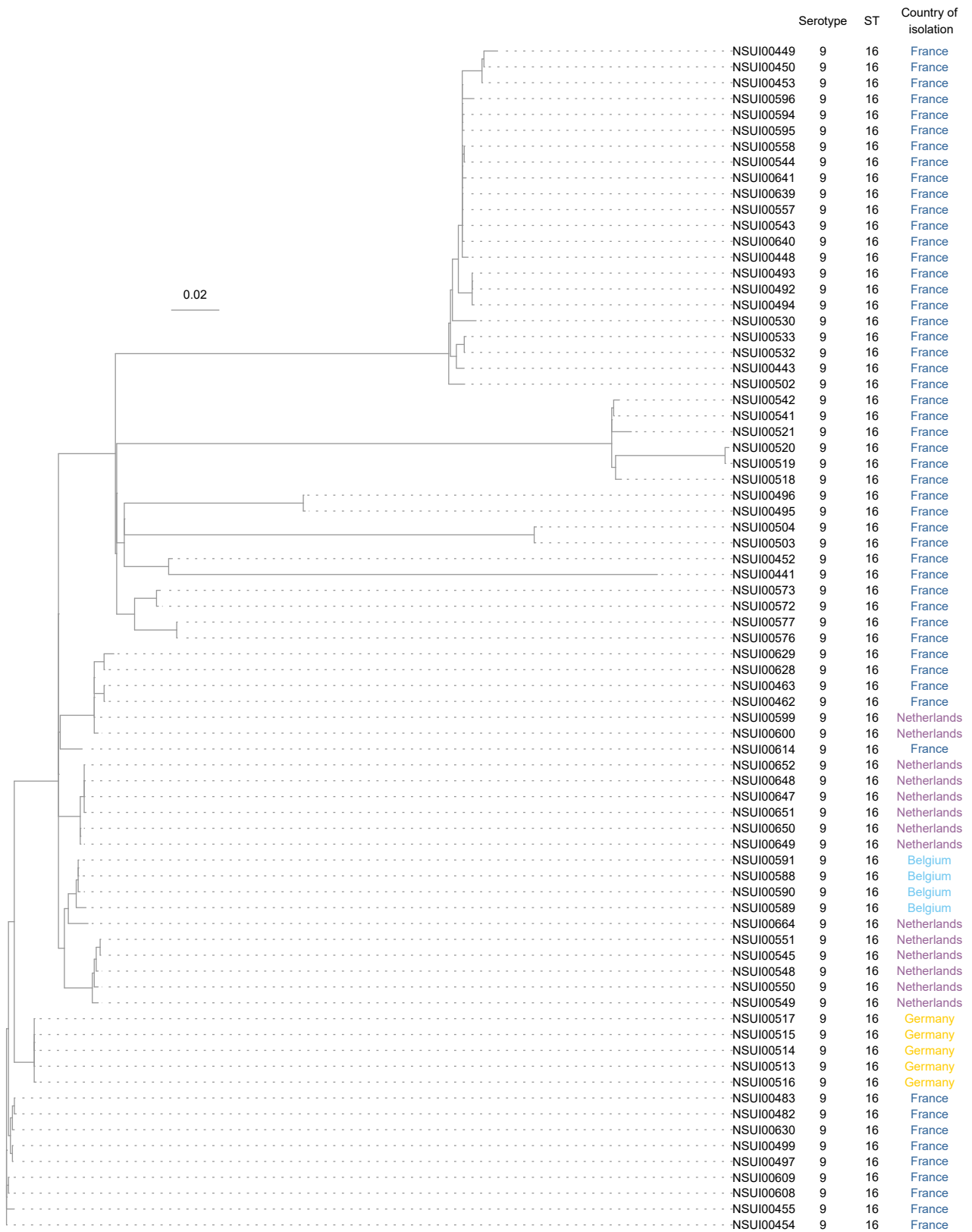

Supplement: Supplementary file 7 — Additional file 7. Inferred genetic relationships based on core-genome analysis among the 75 Streptococcus suis isolates belonging to sequence type 16 investigated in this study. The maximum likelihood phylogenetic tree was constructed from 8906 non-redundant core-genome SNP loci identified relative to the genome sequence of the ST16 serotype 9 reference strain GD-0088. Each isolate is uniquely identified (e.g., NSUI00524) and annotated with its serotype and ST. The tree includes isolates from Belgium, France, Germany, and the Netherlands, with each country represented by a specific color. [file 13567_2024_1366_MOESM7_ESM.pdf]

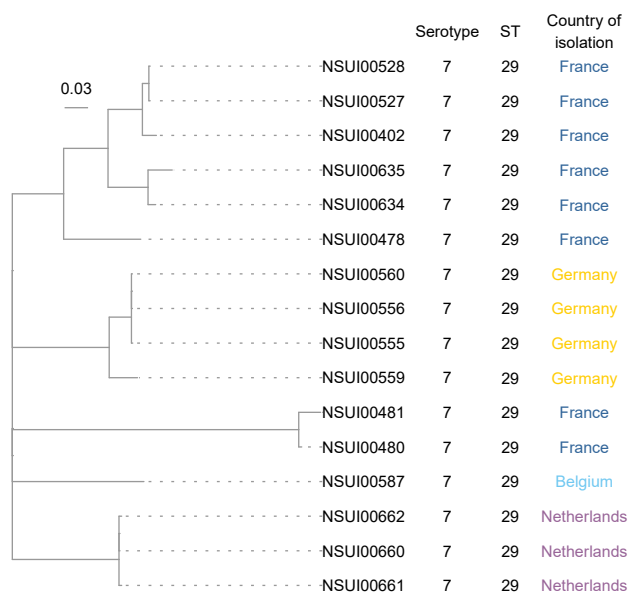

Supplement: Supplementary file 8 — Additional file 8. Inferred genetic relationships based on core-genome analysis among the 16 S. suis isolates belonging to sequence type 29 investigated in this study. The maximum likelihood phylogenetic tree was constructed from 645 non-redundant core-genome SNP loci identified relative to the genome sequence of the ST29 serotype 7 reference strain 13-00283-02 (GenBank Accession number NZ_CP058741.1). Each isolate is uniquely identified (e.g., NSUI00528) and annotated with its serotype and ST. The tree includes isolates from France, Germany, Hungary, and the United Kingdom, with each country represented by a specific color. [file 13567_2024_1366_MOESM8_ESM.pdf]

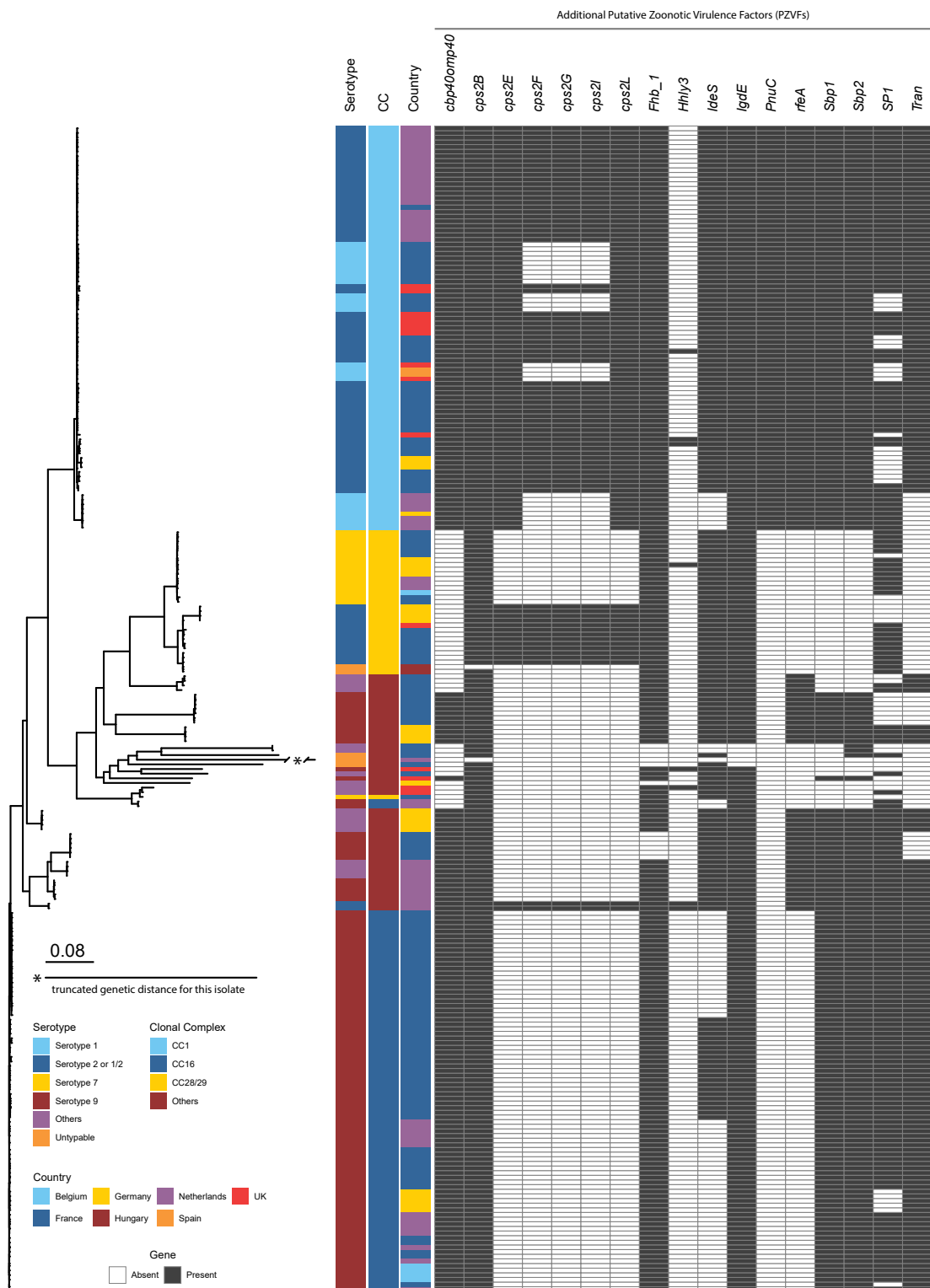

Supplement: Supplementary file 11 — Additional file 11. Detection of 17 additional putative zoonotic virulence factors (PZVFs) among 251 Streptococcus suis isolates. This figure presents a maximum-likelihood phylogenetic tree (left panel), constructed using 8,558 non-redundant core-genome SNP loci identified relative to the genome sequence of the ST1 serotype 2 reference strain P1/7. The right panel depicts general strain information (serotype, clonal complex, country; 3 left-most columns), and PZVFs presence (black) or absence (white) as determined from the whole-genome sequences of each isolate. Isolates of serotypes 1, 2 and 1/2 belonging to CC1 had more PZVFs than other serotypes. [file 13567_2024_1366_MOESM11_ESM.pdf]

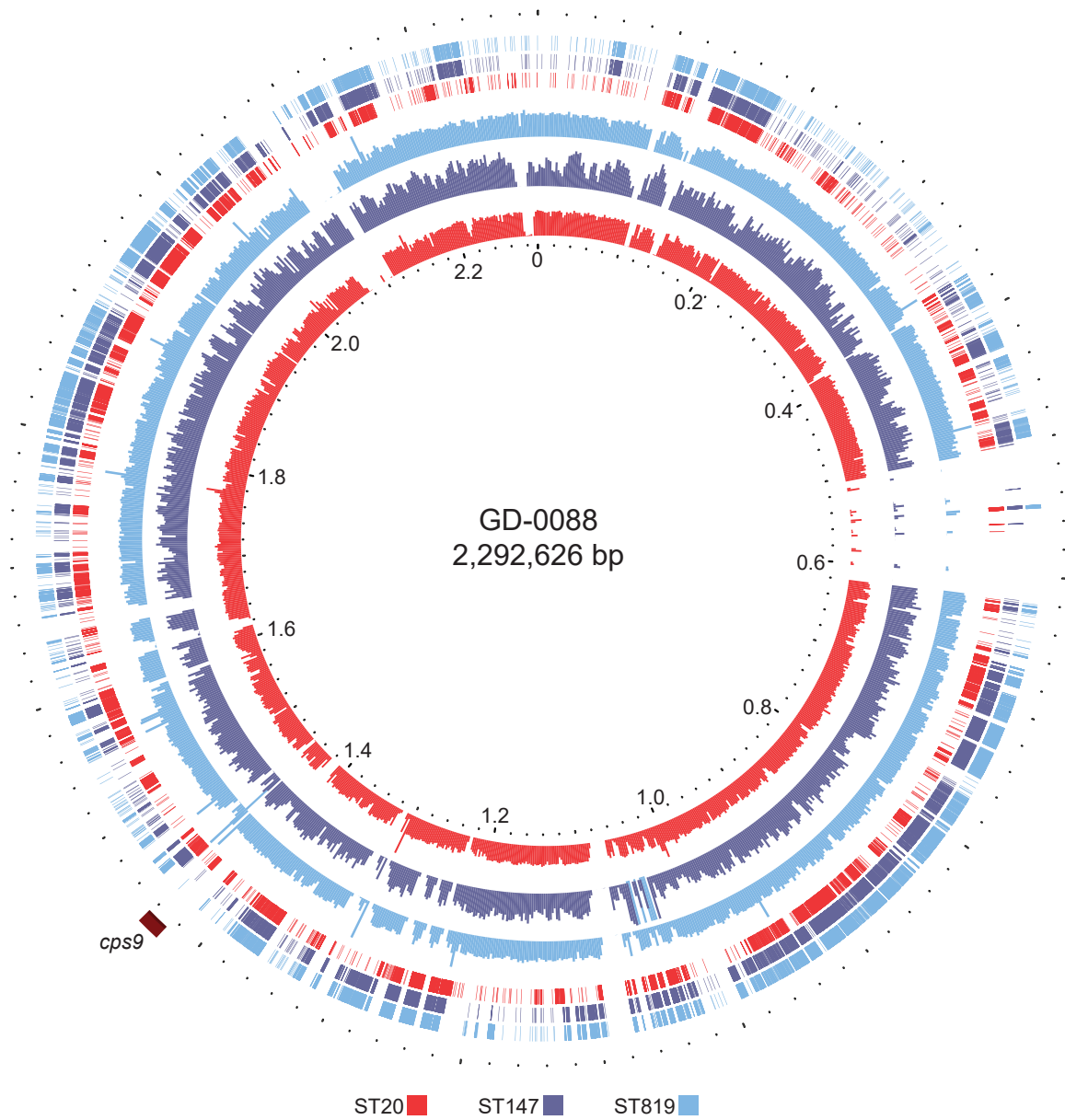

Supplement: Supplementary file 12 — Additional file 12. Genomic synteny of Streptococcus suis ST20, ST147, and ST819 strains in comparison to ST16 serotype 9 reference strain GD-0088. Coverage data (innermost circles) and single-nucleotide polymorphisms (SNPs, outermost circles) for strains NSUI00645 (ST20), NSUI00474 (ST147) and NSUI00682 (ST819) are plotted against the ST16 serotype 9 reference strain GD-0088. The similarity in SNP distribution patterns across several areas of the genome between the ST20, the ST147 and the ST819 isolates does not support the hypothesis that ST20 strains are derived directly from an ST16 organism. There were 13934, 16601, 16113 SNPs for strains NSUI00645, NSUI00474, and NSUI00682, respectively, relative to the reference strains. The position of the cps9 locus in the reference genome is provided as a reference. [file 13567_2024_1366_MOESM12_ESM.pdf]
