# Supplementary material for: Molecular characterization of Streptococcus suis isolates recovered from diseased pigs in Europe
Source: Vet Res. 2024 Sep 27;55:117. doi: 10.1186/s13567-024-01366-y (PMC11429987; doi:10.1186/s13567-024-01366-y)
Supplement: Supplementary file 3 — Additional file 3. STs identified among the Streptococcus suis isolates used in this study. This file contains a table of STs distribution of 251 S. suis isolates used in this study along with their respective housekeeping gene allele number. [file 13567_2024_1366_MOESM3_ESM.docx]

**Additional file 3. Multilocus Sequence Typing (MLST) Sequence Types identified among the *Streptococcus suis* isolates used in this study.**

| **ST^a^** | **Allele number for MLST genes** | | | | | | | **Number of isolates** |
| --- | --- | --- | --- | --- | --- | --- | --- | --- |
|  | ***aroA*** | ***cpn60*** | ***dpr*** | ***gki*** | ***mutS*** | ***recA*** | ***thrA*** |  |
| 1 | 1 | 1 | 1 | 1 | 1 | 1 | 1 | 75 |
| 2 | 1 | 1 | 1 | 2 | 1 | 1 | 1 | 5 |
| 16 | 5 | 17 | 5 | 12 | 1 | 10 | 4 | 75 |
| 17 | 8 | 1 | 5 | 12 | 1 | 10 | 1 | 5 |
| 20 | 1 | 1 | 5 | 12 | 1 | 10 | 1 | 2 |
| 28 | 2 | 30 | 5 | 34 | 31 | 3 | 25 | 15 |
| 29 | 8 | 30 | 5 | 34 | 30 | 3 | 25 | 16 |
| 87 | 18 | 8 | 24 | 12 | 1 | 10 | 4 | 1 |
| 94 | 8 | 21 | 5 | 45 | 44 | 22 | 4 | 1 |
| 108 | 8 | 21 | 5 | 45 | 44 | 22 | 23 | 1 |
| 147 | 5 | 1 | 5 | 12 | 1 | 10 | 1 | 3 |
| 819 | 243 | 1 | 5 | 12 | 1 | 10 | 1 | 2 |
| 977 | 270 | 21 | 5 | 45 | 44 | 22 | 23 | 2 |
| 1222 | 63 | 75 | 21 | 15 | 7 | 30 | 8 | 1 |
| 1508 | 18 | 1 | 5 | 12 | 29 | 10 | 1 | 6 |
| 1520 | 5 | 17 | 5 | 12 | 1 | 292 | 4 | 4 |
| 1521 | 365 | 3 | 5 | 396 | 28 | 21 | 1 | 7 |
| 1552 | 368 | 1 | 1 | 1 | 1 | 1 | 1 | 2 |
| 2753 | 37 | 1 | 1 | 1 | 1 | 1 | 1 | 3 |
| 2757 | 365 | 6 | 18 | 49 | 28 | 7 | 4 | 4 |
| 2760 | 5 | 8 | 5 | 12 | 1 | 10 | 4 | 2 |
| 2767 | 139 | 55 | 97 | 626 | 629 | 91 | 436 | 1 |
| 2768 | 1 | 1 | 1 | 60 | 1 | 6 | 1 | 2 |
| 2769 | 18 | 1 | 5 | 12 | 1 | 10 | 50 | 4 |
| 2771 | 6 | 8 | 510 | 12 | 1 | 10 | 4 | 1 |
| 2772 | 569 | 17 | 5 | 12 | 1 | 10 | 4 | 2 |
| 2773 | 570 | 17 | 5 | 12 | 1 | 10 | 4 | 1 |
| 2774 | 571 | 99 | 21 | 628 | 14 | 6 | 106 | 1 |
| 2775 | 18 | 8 | 24 | 12 | 615 | 10 | 4 | 1 |
| 2790 | 406 | 729 | 354 | 637 | 638 | 473 | 468 | 1 |
| 2791 | 8 | 17 | 514 | 12 | 639 | 481 | 346 | 1 |
| 2793 | 410 | 543 | 36 | 16 | 640 | 38 | 13 | 2 |
| 2796 | 57 | 308 | 40 | 638 | 98 | 15 | 193 | 1 |
| 2798 | 96 | 736 | 515 | 140 | 630 | 38 | 149 | 1 |

^a^ ST: sequence type as determined by *in silico*-based multilocus sequence typing.
