# Supplementary material for: Molecular characterization of Streptococcus suis isolates recovered from diseased pigs in Europe
Source: Vet Res. 2024 Sep 27;55:117. doi: 10.1186/s13567-024-01366-y (PMC11429987; doi:10.1186/s13567-024-01366-y)
Supplement: Supplementary file 9 — Additional file 9. Distribution of classical virulence-associated genes (VAGs) across Streptococcus suis serotypes. This file contains a table of the association of S. suis serotypes and the identified classical virulence-associated gene (mrp, epf, sly) profiles. [file 13567_2024_1366_MOESM9_ESM.docx]

**Additional file 9. Distribution of classical virulence-associated genes (VAGs) across *Streptococcus suis* serotypes.**

| Serotype | Number of isolates | Presence of classical VAG | | | Classical VAG profile | | | | | | |
| --- | --- | --- | --- | --- | --- | --- | --- | --- | --- | --- | --- |
|  |  | *mrp*+ | *epf*+ | *sly*+ | *mrp*+/*epf*+/*sly*+ | *mrp*+/*epf*-/*sly*+ | *mrp*+/*epf*-/*sly*- | *mrp*-/*epf*+/*sly*+ | *mrp*-/*epf*-/*sly*- | *mrp*-/*epf*+/*sly*- | *mrp*+/*epf*+/*sly*- |
| 1 | 25 | 25 | 25 | 25 | 25 | 0 | 0 | 0 | 0 | 0 | 0 |
| 1/2 | 5 | 5 | 3 | 3 | 3 | 0 | 2 | 0 | 0 | 0 | 0 |
| 2 | 72 | 72 | 61 | 61 | 61 | 0 | 11 | 0 | 0 | 0 | 0 |
| 3 | 2 | 2 | 1 | 1 | 1 | 0 | 1 | 0 | 0 | 0 | 0 |
| 4 | 5 | 5 | 5 | 5 | 5 | 0 | 0 | 0 | 0 | 0 | 0 |
| 5 | 2 | 2 | 2 | 2 | 2 | 0 | 0 | 0 | 0 | 0 | 0 |
| 7 | 17 | 17 | 0 | 1 | 0 | 1 | 16 | 0 | 0 | 0 | 0 |
| 8 | 2 | 2 | 0 | 2 | 0 | 2 | 0 | 0 | 0 | 0 | 0 |
| 9 | 108 | 96 | 22 | 107 | 11 | 85 | 0 | 11 | 1 | 0 | 0 |
| 10 | 4 | 0 | 4 | 4 | 0 | 0 | 0 | 4 | 0 | 0 | 0 |
| 16 | 1 | 0 | 0 | 0 | 0 | 0 | 0 | 0 | 1 | 0 | 0 |
| 18 | 2 | 0 | 2 | 0 | 0 | 0 | 0 | 0 | 0 | 2 | 0 |
| 23 | 1 | 1 | 1 | 1 | 1 | 0 | 0 | 0 | 0 | 0 | 0 |
| UT^a^ | 5 | 4 | 1 | 0 | 0 | 0 | 3 | 0 | 1 | 0 | 1 |
| Total | 251 | 231 | 127 | 212 | 109 | 88 | 33 | 15 | 3 | 2 | 1 |

^a^UT: Untypable.
