# Supplementary material for: Molecular characterization of Streptococcus suis isolates recovered from diseased pigs in Europe
Source: Vet Res. 2024 Sep 27;55:117. doi: 10.1186/s13567-024-01366-y (PMC11429987; doi:10.1186/s13567-024-01366-y)
Supplement: Supplementary file 10 — Additional file 10. Detection of virulence-associated genes (VAGs) in the Streptococcus suis isolates used in this study. This file contains a list of 80+ VAGs screened in each S. suis strains in this study, including differential presences in different sequence types. [file 13567_2024_1366_MOESM10_ESM.docx]

**Additional file 10. Detection of virulence-associated genes (VAGs) in the *Streptococcus suis* isolates used in this study.**

| Description | Virulence associated genes (VAGs) |
| --- | --- |
| Catalog of additional VAGs used to interrogate the genomes of the *Streptococcus suis* isolates^a^ | *treR, SSU05_1311, ihk, irr, ofs, adcR, sadP, fur, lmb, luxS, SSU0501, dltA, SMU-61-like, zur, lspA, sspA, strA, ciaR, hyl, atl, ssa, covR, sao, feoB, pgdA, virA, scrR, ssnA, srtB, srtC, srtD, srtF, rgg, HP0245, SSU05_0473, neuB, SpyM3-0908, SSU0835, Endo D, revS, nadR, ABC, srtG, salK, salR, virB4, virD4, CDS2157, stp, ssads, EIIAB, serS, purD, glnA, DPPIV, SSU207, gdh, htps, yhbU/orf207, SSU0458, arcA, igA1, cdd, ciaH, fbps, sodA, gtfA, lgt, dpr, gndA, manN, scrB, purA, apuA, troA, guaB, tig, GAPDH, guaA, ccpA, eno, gpmA, nisK, nisR* |
| Additional VAGs (*N*=35) present in all isolates^b^ | *CDS2157, stp, ssads, EIIAB, serS, purD, glnA, DPPIV, SSU207, gdh, htps, yhbU/orf207, SSU0458, arcA, igA1, cdd, ciaH, fbps, sodA, gtfA, lgt, dpr, gndA, manN, scrB, purA, apuA, troA, guaB, tig, GAPDH, guaA, ccpA, eno, gpmA* |
| Additional VAGs (*N*=76) present in all ST1 isolates | *treR, SSU05_1311, ihk, irr, ofs, adcR, sadP, fur, lmb, luxS, SSU0501, dltA, SMU-61-like, zur, lspA, sspA, strA, ciaR, hyl, atl, ssa, covR, sao, feoB, pgdA, virA, scrR, ssnA, srtB, srtC, srtD, srtF, rgg, HP0245, SSU05_0473, neuB, SpyM3-0908, SSU0835, Endo D, revS, nadR*, plus the 35 VAGs present in all isolates |
| Additional VAGs (*N*=8) absent from all ST1 isolates | *ABC, srtG, salK, salR, virB4, virD4,* plus *nisK*, and *nisR* ^b^ |
| Additional VAGs (*N*=67) present in all ST28 isolates | *treR, SSU05_1311, ihk, irr, ofs, adcR, sadP, fur, lmb, luxS, SSU0501, dltA, SMU-61-like, zur, lspA, sspA, strA, ciaR, hyl, atl, ssa, covR, sao, feoB, pgdA, virA, scrR, ssnA, srtF, HP0245, SSU05_0473, srtG,* plus the 35 VAGs present in all isolates |
| Additional VAGs (*N*=16) absent from all ST28 isolates | *srtB, srtC, srtD, rgg, SpyM3-0908, SSU0835, Endo D, revS, nadR, ABC, salK, salR, virB4, virD4,* plus *nisK* and *nisR* ^b^ |
| Additional VAGs (*N*=67) present in all ST29 serotype 7 isolates | *treR, SSU05_1311, ihk, irr, ofs, adcR, sadP, fur, lmb, luxS, SSU0501, dltA, SMU-61-like, zur, lspA, sspA, strA, ciaR, hyl, atl, ssa, covR, sao, feoB, pgdA, virA, scrR, ssnA, srtF, rgg, HP0245, SSU05_0473,* plus the 35 VAGs present in all isolates |
| Additional VAGs (*N*=15) absent from all ST29 serotype 7 isolates | *srtB, srtC, srtD, neuB, SpyM3-0908, SSU0835, Endo D, revS, nadR, ABC, srtG, salK, salR,* plus *nisK,* and *nisR*^b^ |
| Additional VAGs (*N*=58) present in all serotype 9 isolates | *treR, SSU05_1311, ihk, ofs, adcR, fur, lmb, luxS, dltA, zur, lspA, sspA, strA, ciaR, atl, ssa, covR, sao, feoB, pgdA, scrR, ssnA, Endo D,* plus the 35 VAGs present in all isolates |
| Additional VAGs (*N*=5) absent from all serotype 9 isolates | *neuB, nadR, srtG,* plus *nisK*, and *nisR* ^b^ |
| Additional VAGs (*N*=66) present in all ST16 serotype 9 isolates | *treR, SSU05_1311, ihk, ofs, adcR, sadP, fur, lmb, luxS, dltA, zur, lspA, sspA, strA, ciaR, hyl, atl, ssa, covR, sao, feoB, pgdA, virA, scrR, ssnA, srtB, srtC, srtD, HP0245, Endo D, revS*, plus the 35 VAGs present in all isolates |
| Additional VAGs (*N*=14) absent from all ST16 serotype 9 isolates | *srtF, rgg, SSU05_0473, neuB, SpyM3-0908, SSU0835, nadR, ABC, srtG, salK, salR,* plus *nisK*, and *nisR* ^b^ |

^a^ We also screened for the presence of the classical VAGs *mrp*, *epf* and *sly* (see Additional file 3).

^b^ Genes *nisK* and *nisR* were absent from all isolates.
